# Typology of Deflation-Corrected Estimators of Reliability

**DOI:** 10.3389/fpsyg.2022.891959

**Published:** 2022-07-18

**Authors:** Jari Metsämuuronen

**Affiliations:** ^1^Finnish Education Evaluation Centre, Helsinki, Finland; ^2^Centre for Learning Analytics, University of Turku, Turku, Finland

**Keywords:** reliability, deflation-corrected reliability, deflation in reliability, coefficient alpha, coefficient theta, coefficient omega, maximal reliability

## Abstract

The reliability of a test score is discussed from the viewpoint of underestimation of and, specifically, deflation in estimates or reliability. Many widely used estimators are known to underestimate reliability. Empirical cases have shown that estimates by widely used estimators such as alpha, theta, omega, and rho may be deflated by up to 0.60 units of reliability or even more, with certain types of datasets. The reason for this radical deflation lies in the item–score correlation (*Rit*) embedded in the estimators: because the estimates by *Rit* are deflated when the number of categories in scales are far from each other, as is always the case with item and score, the estimates of reliability are deflated as well. A short-cut method to reach estimates closer to the true magnitude, new types of estimators, and deflation-corrected estimators of reliability (DCERs), are studied in the article. The empirical section is a study on the characteristics of combinations of DCERs formed by different bases for estimators (alpha, theta, omega, and rho), different alternative estimators of correlation as the linking factor between item and the score variable, and different conditions. Based on the simulation, an initial typology of the families of DCERs is presented: some estimators are better with binary items and some with polytomous items; some are better with small sample sizes and some with larger ones.

## Introduction

### From Parallel Test Reliability to Alpha and Maximal Reliability and Beyond From the Perspective of Underestimation in Estimates

*Reliability has often been underestimated by the conventional formula […]. Many tests are more reliable than they have been considered to be* (Guttman, 1945, p. 260.).

The reliability of a test score generated by a compilation of multiple test items has interested scholars for more than 100 years. In the early phase of the history of measurement modeling, the interest shifted from measurement error to reliability, although measurement error may be a more profound concept than reliability (Gulliksen, 1950). Ever since reliability has become a central measure used to quantify the amount of a random measurement error that exists in a test score. These two concepts are closely linked though because the standard error of the measurement $S.E.m=\sigma_{E}=\sigma_{X}\sqrt{1-REL}$ is defined by reliability $REL=\sigma_{T}^{2}/\sigma_{X}^{2}=1-\sigma_{E}^{2}/\sigma_{X}^{2}$ (e.g., Gulliksen, 1950), where $\sigma_{T}^{2}$, $\sigma_{X}^{2}$, and refers to the variances of the observed score variable (*X*), unobserved true score (*T*), and error element (*E*) familiar from their profound relation in testing theory, *X* = *T* + *E*. Because the true score *T* is unobservable, the error element *E* is also unobservable; therefore, several measurement models based on parallel, tau-equivalent, and congeneric partitions of the test or test items (referring to, e.g., Lord et al., 1968) with different assumptions and multiple estimators of reliability have been developed over the years.

It is well-known that many estimators of reliability underestimate population reliability because of the *attenuation* caused by errors in measurement modeling and random errors in the measurement. However, a less-discussed issue regarding estimates by traditional estimators of reliability is that the estimates may also be radically *deflated* because of artificial systemic errors during the estimation. These concepts are discussed, for instance, by Chan (2008), Lavrakas (2008), Gadermann et al. (2012), Revelle and Condon (2018), and Metsämuuronen (2022a,c,f). Deflation and its correction are the main foci in this article. Some historical turning points and traditional estimators of reliability are discussed from the viewpoint of underestimation in reliability to lead the focus from traditional estimators to the deflation-corrected estimators of reliability discussed in the latter part of the article.

### From Brown and Spearman to the Greatest Lower Bound of Reliability

First traces of reliability lead us to Brown (1910) and Spearman (1910), who suggested a way to correct attenuation in the product-moment correlation coefficient (PMC; Bravais, 1844; Pearson, 1896 onward). Pearson (1903) had already noticed that when only a portion of the range of a variable's values is actualized in the sample, this leads to inaccuracy in the estimates of correlation; the estimates are attenuated. This phenomenon is often discussed as range restriction or restriction of range (refer to the literature, e.g., Sackett and Yang, 2000; Sackett et al., 2007; Schmidt et al., 2008; Schmidt and Hunter, 2015). Pearson (1903) and Spearman (1904) were the first to offer solutions to the problem. Later, a coefficient of reliability, the Brown–Spearman prediction formula of reliability based on strictly parallel tests [ρ_BS_; refer to Cho and Chun (2018) for the history and rationale of the rectified order of innovators], was famously developed to correct the inaccuracy in correlation first by Brown in his unpublished doctoral thesis [before 1910 although referred to in Brown (1910) and later in Spearman (1910). ρ_BS_ is based on a correlation between the strictly parallel partitions *g* and *h* of a test. Parallelism implies that the true scores (taus) and variances of a test-taker are assumed to be equal in the sub-tests [*T*_*g*_ = *T*_*h*_, $\sigma_{g}^{2}=\sigma_{h}^{2}$; refer to Gulliksen (1950)].

A more useful early innovation based on two partitions, *g* and *h*, was offered by Rulon (1939) after being consulted by Flanagan (see the history in Cho and Chun, 2018) based on tau-equivalent partitions: although the lengths of partitions *g* and *h* should be equal, they need not be strictly parallel; that is, although the true values of a test-taker are assumed to be (essentially) equal, the variances in the partitions need not be equal (*T*_*g*_ = *T*_*h*_, $\sigma_{g}^{2}\neq \sigma_{h}^{2}$). The form of the Flanagan–Rulon prediction formula (ρ_FR_) appears to be the same as ρ_BS_, or the form of ρ_BS_ can be expressed in the form of ρ_FR_(refer to Lord et al., 1968), but the less strict assumptions led to a useful application in the form of the coefficient alpha that will be discussed later. Later, both ρ_BS_ and ρ_FR_ were shown by Guttman (1945) to underestimate population reliability.

Guttman (1945) was the first to show the technical or mechanical basis for underestimation in reliability. All of his six coefficients of reliability (λ_1_ − λ_6_) were shown to underestimate the true population reliability. Of these, λ_3_ and λ_4_ appear to be important from the general viewpoint, with λ_4_ being a general case of ρ_BS_ and ρ_FR_ and λ_3_ being equal to the coefficient alpha that will be discussed later. λ_4_ was shown to underestimate reliability “*no matter how the test is split*” (Guttman, 1945, p. 260, emphasis original); hence, both ρ_BS_ and ρ_FR_ underestimate the population reliability. The same also applies to an estimator called the greatest lower bound of reliability (ρ_GLB_) based on λ_4_ suggested already by Guttman (1945) and studied later, among others, by Jackson and Agunwamba (1977), Woodhouse and Jackson (1977), and Ten Berge et al. (from Ten Berge and Zegers, 1978 onward; Revelle, 2015; refer also to e.g., Moltner and Revelle, 2015; Trizano-Hermosilla and Alvarado, 2016). Also, McDonald's hierarchical omega (ρ_ω*H*_; McDonald, 1999) and Revelle's β (Revelle, 1979; refer also to Zinbarg et al., 2005; Revelle and Zinbarg, 2009) is based on the idea of the *lowest* lower bound of reliability (ρ_LLB_) belonging to this family [refer to the comparison of estimators based on different types of partition in Revelle (2021) and simulation in Edwards et al. (2021)]. While all the estimators ρ_BS_, ρ_FR_, and ρ_GLB_ underestimate the population reliability (ρ_*population*_), estimators in the framework of ρ_LLB_ give *obvious* underestimations. From the underestimation viewpoint, their relationship is then as follows:


(1) $$\begin{array}{l} \rho_{LLB}<\rho_{FR}\leq \rho_{BS}\leq \rho_{GLB}<\rho_{population}. \end{array}$$

### From Prediction Formulae to Coefficient Alpha

Even before the Flanagan–Rulon formula, Kuder and Richardson (1937) had generalized the idea initiated by Brown and Spearman to a form where each test item in a compilation was taken either as a parallel partition (leading to the coefficient known as KR21, ρ_KR21_) or a non-parallel although tau-equivalent (or “essentially” tau-equivalent, refer Novick and Lewis, 1967) partition of the test (KR20, ρ_KR20_). The latter appeared to be more useful in practical testing settings, and it is still in wide use with binary items as one of the lower bounds of reliability.

While KR20 was derived for binary items, the formula was soon generalized to also allow polytomous items (the first usage seems to be in Jackson and Ferguson, 1941; refer to Cho and Chun, 2018), and it was later named coefficient alpha (ρ_α_) by Cronbach (1951). Cronbach showed that the estimate by ρ_α_ is the mean of all split-half partitions (Cronbach, 1951; refer to other interpretations in Cortina, 1993). Warrens (2015) reminds us, though, that this holds only (a) when the partitions include the same number of items, which implies that (b) there are an even number of items on the test to form split-halves with an equal number of items, and (c) when the Flanagan–Rulon formula instead of the Brown–Spearman formula is used.

Because ρ_KR20_, ρ_KR21_, and ρ_α_ are special cases of Guttman's λ_3_, they all underestimate the population reliability. Errors in measurement modeling^1^ and attenuation have been approximated to cause an underestimation of the magnitude of around 1–11% (see Raykov, 1997a; Graham, 2006; Green and Yang, 2009a; Trizano-Hermosilla and Alvarado, 2016). However, it is generally accepted that when all items are (essentially) tau-equivalent, the phenomenon is unidimensional, and the item-wise errors do not correlate; these estimators would reflect the true reliability (refer to Novick and Lewis, 1967; refer to discussion in, e.g., Cheng et al., 2012; Raykov and Marcoulides, 2017). Unfortunately, this seems to be true only when it comes to *attenuation* in the estimates; this is not true for *deflation*, because the calculation process itself includes a technical or mechanical error that causes deflation in the estimates. The root cause of deflation in ρ_α_ is the deflation in item–score correlation (ρ_*iX*_, *Rit*) embedded in the estimators of reliability; item–score correlation is shown to be severely deflated in settings related to measurement modeling where the scales of the variables deviate radically from each other [refer to algebraic reasons in Metsämuuronen (2016, 2017) and simulations in Metsämuuronen (2020a,b, 2021a, 2022b)]. This element is visible in the form of ρ_α_ provided in Lord et al. (1968):


(2) $$\begin{array}{l} \rho_{\alpha }=\frac{k}{k-1}\left(1-\frac{\overset{k}{\underset{i\;=\;1}{\sum }}\sigma_{i}^{2}}{{\left(\overset{k}{\underset{i\;=\;1}{\sum }}\sigma_{i}\rho_{iX}\right)}^{2}}\right), \end{array}$$

where *k* is the number of items in the compilation and $\sigma_{i}^{2}$ refers to the variance of item *g*_*i*_. Because of thisρ_*iX*_, the estimates of reliability by coefficient alpha may be deflated to the extent of 0.6 units (refer to examples of this magnitude in, e.g., Zumbo et al., 2007; Gadermann et al., 2012; Metsämuuronen and Ukkola, 2019; Metsämuuronen, 2022a,c). Then, from the underestimation viewpoint, the relationship of these estimators is as follows:


(3) $$\begin{array}{l} \rho_{KR20}\leq \rho_{KR21}=\rho_{\alpha }<<\rho_{population}. \end{array}$$

Despite the known characteristic to underestimate reliability, ρ_α_ is the most used estimator of reliability in real-life test settings (refer to literature in, e.g., Hoekstra et al., 2019), most probably because of its computational simplicity and obvious conservative nature (e.g., Metsämuuronen, 2017). Because of its wide popularity, alpha has been said to be the *most often wrongly understood* statistic (refer to discussion in, e.g., Sijtsma, 2009; Cho and Kim, 2015; Hoekstra et al., 2019). Therefore, many scholars are ready to remove ρ_α_ from use (refer to the discussion in, e.g., Sijtsma, 2009; Yang and Green, 2011; Dunn et al., 2013; Trizano-Hermosilla and Alvarado, 2016; McNeish, 2017). However, the issue is still far from settled. Among others, Bentler (2009); Falk and Savalei (2011); Raykov et al. (2014); Metsämuuronen (2017); Raykov and Marcoulides (2017), seem to share stand that when its assumptions are understood and met, ρ_α_ may be a useful simple tool for assessing (one of) the lower bound(s) of reliability of the score in real-life testing settings. Maybe what is more problematic in the use of ρ_α_ is that many scholars who use ρ_α_ may not be able to name *any other* coefficient of reliability that they can use instead. In an empirical study by Hoekstra et al. (2019), 23% of the researchers who published their results in selected renowned journals fell in this group.

### From Alpha to Theta, Omega, and Maximal Reliability

The least restricted family of measurement models is based on congeneric partitions of the test. In these models, the true values of the same test-taker need not be identical in the partitions, which means that the assumption of equally long partitions and the same scale in the test items is not required. Also, the weights of items or partitions need not be equal, which allows for multidimensionality in the phenomenon, or the measurement errors, and they need not be independent of each other, too.

Many coefficients of reliability have been developed for these settings. For two congeneric partitions, as counterparts for ρ_BS_ and ρ_FR_, we have estimators by Angoff and Feldt (ρ_AF_; Angoff, 1953; Feldt, 1975), Horst (ρ_H_; Horst, 1951), and Raju (ρ_β_; Raju, 1977). Because the formulae of ρ_AF_ and ρ_β_ include the same estimate of population variance as in ρ_α_: $\sigma_{X}^{2}={\left(\overset{k}{\underset{i\;=\;1}{\sum }}\sigma_{i}\rho_{iX}\right)}^{2}$, these estimators also tend to give deflated estimates, because the estimate of the item–score correlation byρ_*iX*_ is deflated. Based on Warrens (2016), the proportional tendency of these estimators is as follows: if the partitions are equally long, the magnitude of the estimates gets the relationship


(4) $$\begin{array}{l} \rho_{FR}=\rho_{\beta }\leq \rho_{SB}=\rho_{H}\leq \rho_{AF}<<\rho_{population}, \end{array}$$

that is, if the condition optimal for ρ_AF_ is fulfilled, other estimators tend to underestimate reliability, and all estimators may produce deflated estimates where the magnitude of the deflation depends on several characteristics such as the difficulty levels of the items. If the variances of the partitions are equal, then


(5) $$\begin{array}{l} \rho_{FR}=\rho_{SB}=\rho_{AF}\leq \rho_{H}=\rho_{\beta }<<\rho_{population}, \end{array}$$

that is, if the condition optimal for ρ_H_ and ρ_β_ is fulfilled, other estimators tend to underestimate reliability, and all may be radically deflated.

As counterparts to ρ_α_ for the case in which the scales in items differ from each other, we have two main estimators. For raw scores, we have the Gilmer–Feldt coefficient (ρ_GF_; Gilmer and Feldt, 1983), also known as the Feldt–Raju coefficient (e.g., Feldt and Brennan, 1989) or the Feldt–Gilmer coefficient (e.g., Kim and Feldt, 2010). Instead of number items (refer to eq. 2), ρ_GF_ uses the proportional weight of the items as a calibrating factor in estimation. The estimates by ρ_α_ tend to be mildly lower than those by ρ_GF_. However, the formula of ρ_GF_ uses the same estimate of population variance $\sigma_{X}^{2}={\left(\overset{k}{\underset{i\;=\;1}{\sum }}\sigma_{i}\rho_{iX}\right)}^{2}$as ρ_α_ leading to deflated estimates.

Another alternative for ρ_α_ is to standardize the items and score by principal component analysis (Guttman, 1941), which leads to coefficient theta [ρ_TH_; Kaiser and Caffrey (1965), based on Lord, 1958], also known as Armor's theta (Armor, 1973). While ρ_α_ uses raw scores and observed values in items, ρ_TH_ uses standardized items and scores, which has an advantage over ρ_α_: the principal component score is one of the “optimal linear combinations” of the score discussed over the years by, chronologically, e.g., Thompson (1940), Guttman (1941), Stouffer (1950), Lord (1958), and Bentler (1968). Zumbo et al. (2007), Gadermann et al. (2012), and Metsämuuronen (2022a,c) have brought ρ_TH_ into discussions again: Zumbo and colleagues because of a new type of estimator called ordinal theta and Metsämuuronen as one of the bases for deflation-corrected estimators of reliability discussed later.

Coefficient theta can be expressed as:


(6) $$\begin{array}{l} \rho_{TH}=\frac{k}{k-1}\left(1-\frac{1}{\overset{k}{\underset{i\;=\;1}{\sum }}\lambda_{i\theta }^{2}}\right), \end{array}$$

where λ_*i*θ_ is the principal component loadings of the principal component of a one-latent variable model (or of the first principal component), that is, correlations between items and the score variable. It is known that ρ_TH_ maximizes ρ_α_ (Greene and Carmines, 1980). This can be partly explained by a more effective formula and partly by a more optimally constructed score variable (raw score vs. principal component score). Empirical findings indicate that ρ_TH_ also tends to be conservative (Metsämuuronen, 2022a,f); that is, it seems to underestimate the population reliability although less than the alpha and omega do; the latter will be discussed later. From the viewpoint of underestimation, the relationship of these estimators is then:


(7) $$\begin{array}{l} \rho_{\alpha }\leq \rho_{GF}<\rho_{TH}<\rho_{population}. \end{array}$$

In the recent decades, much effort has been gone to explore different aspects of estimators of reliability within the framework of factor models or, more generally, within the latent variable modeling (of the models, refer to, e.g., McDonald, 1985, 1999; Raykov and Marcoulides, 2010). Two of the most discussed estimators are coefficient omega total (ρ_ω_; later, just omega), based on the studies of Heise and Bohrnstedt (1970) and McDonald (1970, 1999), and coefficient rho or maximal reliability (ρ_MAX_; for instance, Raykov, 1997b, 2004), also known as Raykov's rho (refer to, e.g., Cleff, 2019) and Hancock's H (Hancock and Mueller, 2001), based on the conceptualization of “optimal linear combination” discussed above, and later unified by Li et al. (1996) and Li (1997). The two estimators are based on conventions related to factor analysis and factor loadings (λ_*i*θ_). An ancestor of this family is ρ_TH_, which is based on the principal component analysis discussed above.

Coefficient omega can be expressed as follows:


(8) $$\begin{array}{l} \rho_{\omega }=\frac{{\left(\overset{k}{\underset{i\;=\;1}{\sum }}\lambda_{i\theta }\right)}^{2}}{{\left(\overset{k}{\underset{i\;=\;1}{\sum }}\lambda_{i\theta }\right)}^{2}+\overset{k}{\underset{i\;=\;1}{\sum }}\left(1-\lambda_{i\theta }^{2}\right)}, \end{array}$$

and rho as:


(9) $$\begin{array}{l} \rho_{MAX}=\frac{1}{1+\frac{1}{\overset{k}{\underset{i\;=\;1}{\sum }}\left(\lambda_{i\theta }^{2}/\left(1-\lambda_{i\theta }^{2}\right)\right)}}, \end{array}$$

where λ_*i*θ_ refers to factor loadings by maximum likelihood estimation of a one-latent variable model, although models with multiple dimensions are also in use. The measurement model related to these estimators will be discussed later.

In the theoretical case where all item weights are equal, ρ_TH_, ρ_ω_, and ρ_MAX_ are equal to ρ_α_. From this viewpoint, it may be correct to conclude that ρ_TH_, ρ_ω_, and ρ_MAX_ are general forms of ρ_α_ (refer to, e.g., Hayes and Coutts, 2020). Otherwise, the magnitude of the estimates by ρ_α_ is smaller than by ρ_TH_ (Greene and Carmines, 1980), and the magnitude of the estimates by ρ_ω_ is smaller than by ρ_MAX_ (e.g., Cheng et al., 2012). Hence, it seems that both ρ_α_ and ρ_ω_ tend to underestimate reliability. A possible confounding phenomenon is that the estimates of reliability by ρ_MAX_ tend to be overestimated with finite or small sample sizes (refer to Aquirre-Urreta et al., 2019; Metsämuuronen, 2022a,c,f). This is caused by the fact that even if only one item has loading λ_*i*_ ≈ 1, the element $\lambda_{i}^{2}/\left(1-\lambda_{i}^{2}\right)$ in eq. (9) becomes unstable and gives, most probably, a value too high compared to the population. This may happen easily with small sample sizes because they are prone to produce deterministic or near-deterministic patterns of the item–score relationship (see discussion in Metsämuuronen, 2022c,f). From the viewpoint of underestimation, in practical settings excluding the theoretical case of identical factor loadings, the relationship of these estimators is then:


(10) $$\begin{array}{l} \rho_{\alpha }<\rho_{\omega }<\rho_{TH}<\rho_{MAX}<\rho_{population}\left(<\rho_{MAX}\right). \end{array}$$

In real-life settings, the difference between the estimates by ρ_α_, ρ_*TH*_, ρ_ω_, and ρ_MAX_ may be subtle. For example, in a simulation with 1,440 real-life datasets (Metsämuuronen, 2022f), the average magnitude of the lowest estimates by ρ_α_ was 0.024 units of reliability (2.9%) lower than the highest estimates by ρ_*MAX*_. Similarly, the average estimate byρ_ω_ was 0.021 units (2.4 %) lower than by ρ_*MAX*_ and 0.017 units (1.9 %) lower than by ρ_*TH*_. Notably, though, the difference between ρ_α_ and ρ_*MAX*_ seems to be the wider the smaller the sample size is. In the simulation (Metsämuuronen, 2022f), with a sample size of *n* = 25, the average difference between ρ_α_ and ρ_*MAX*_ was 0.056 units of reliability (6.4 %), and with *n* = 200, the difference was just 0.008 units of reliability (0.92 %).

### From Alpha, Theta, Omega, and Rho to Deflation-Corrected Reliability

**Figure 1:**
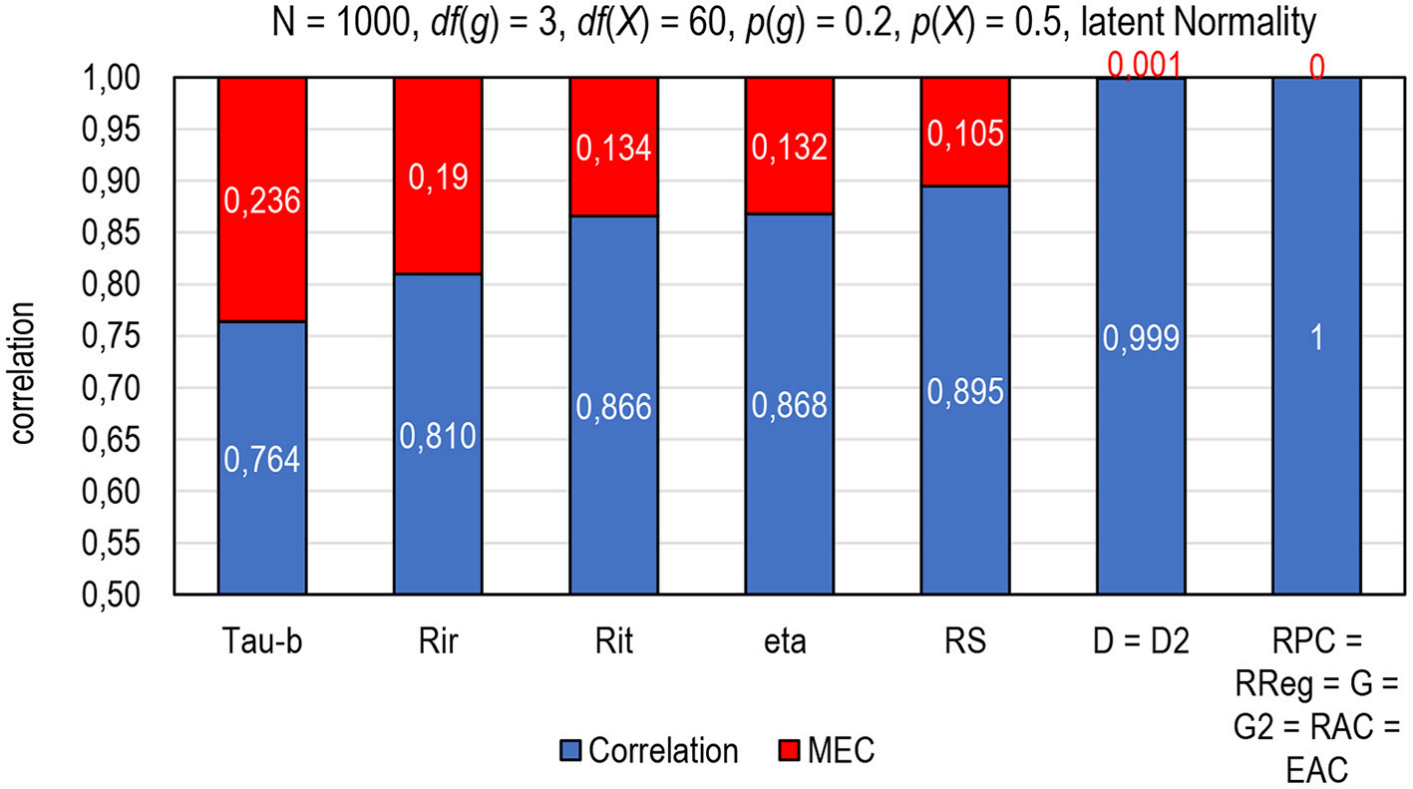
The magnitude of a mechanical error on the estimates of correlation (MEC) by selected estimators of correlation. Tau-b = Kendall tau-b; Rir = Henrysson item–rest correlation ( = PMC), Rit = item–total correlation (= PMC); eta = coefficient eta (X dependent), RS = Spearman rank-order correlation (= PMC), D = Somers delta (X-dependent); D2 = dimension-corrected D; RPC = polychoric correlation; R_REG_ = r-polyreg correlation; G = Goodman-Kruskal gamma; G2 = dimension-corrected G, RAC = attenuation-corrected Rit, EAC = attenuation-corrected eta.

While ρ_α_ is known to underestimate reliability, it seems that ρ_TH_, ρ_ω_, and ρ_MAX_ also tend to give obvious underestimates with certain kinds of datasets, typically with tests of extreme difficulty levels or with incremental difficulty levels including both very easy and very difficult test items. This is a reasonable conclusion from the known character of PMC embedded in the traditional estimators of reliability in the form of *Rit* and λ_*i*_ to underestimate the true correlation when the scales of two variables are far from each other as is typical with an item and the score variable (e.g., Metsämuuronen, 2022a,c,f; refer later to Figure 1). Recall the relationship between PMC = ρ_*gX*_ = *Rit* and the principal component loading (in ρ_TH_) and factor loading (in ρ_ω_ and ρ_MAX_): the loading λ_*i*_ is, essentially, a correlation between an item and a score variable (e.g., Cramer and Howitt, 2004; Yang, 2010).

Knowing that PMC is always deflated in cases where scales in the variables are not equal, as is always the case between an item and the score variable, all the estimators mentioned above are deflated, sometimes radically. Empirical findings show that the estimates by ρ_α_, ρ_*TH*_, ρ_ω_, and ρ_*MAX*_ may be deflated by 0.4–0.6 units of reliability or 46–71% as discussed above (refer to examples in, e.g., Zumbo et al., 2007; Gadermann et al., 2012; Metsämuuronen and Ukkola, 2019; Metsämuuronen, 2022a,c,f). Metsämuuronen (2022a) notes that deflation of this size is remarkable and needs to be studied because it is no more caused by an error in the measurement modeling such as violations in tau-equivalency, unidimensionality, or uncorrelated errors as is traditionally suggested (refer to above). From this point of view, the deflation of 0.4–0.6 units of reliability must be explained directly by some mechanical reasons, and this raises the issue of underestimation in reliability to a new level.

Metsämuuronen (e.g., 2022a; 2022b; 2022f) has used the concept of “mechanical error in the estimates of correlation” (MEC) to understand deflation. The obvious and grave deflation in traditional estimators of reliability has motivated the development of and studies on new types of estimators of reliability called MEC-corrected estimators of reliability (MCERs; Metsämuuronen, 2022a,f) and attenuation-corrected estimators of reliability (ACERs, Metsämuuronen, 2022c), which are both called deflation-corrected estimators of reliability (DCERs; Metsämuuronen, 2022a,f). In MCERs, the embedded *Rit* and λ_*i*_ are replaced by totally *different* estimators of correlation, while in ACERs, *Rit* and λ_*i*_ are replaced by *attenuation-corrected* estimators of correlation. The logic for and forms of these estimators are discussed in Metsämuuronen (2022a), and these will be briefly discussed later. Notably, the ordinal alpha and ordinal theta by Zumbo et al. (2007; refer also to Gadermann et al., 2012) may be included as part of the extended family of DCERs, as, instead of changing the item–score correlation itself, the inter-item matrices of PMCs are replaced by matrices of polychoric correlation coefficients.

From the attenuation and deflation viewpoint, in general, the relationship of these estimators is


(11) $$\begin{array}{l} \rho_{\alpha }<\rho_{\omega }<\rho_{TH}<\rho_{MAX}<<\rho_{DCER}<\rho_{population}. \end{array}$$

Notably, though, certain DCERs based on rho may be prone to overestimating the population reliability with small sample sizes, because rho itself tends to overestimate reliability with small sample sizes (refer to Aquirre-Urreta et al., 2019), while other DCERs based on alpha, theta, and omega, as being more conservative, may be prone to underestimation (see Metsämuuronen, 2022f). This area is largely unstudied, and the current study intends to shed some light on this issue.

Except for the more established coefficient by Zumbo et al. (2007), studies concerning estimators from the family of DCERs are either at a very initial stage (e.g., Metsämuuronen, 2016, 2018), or they give some examples only of the new possibilities (Metsämuuronen, 2020a,b, 2021a,b, 2022b), or they are based on small example datasets and are fragmentary (refer to Metsämuuronen, 2022a,c,f). The simulations by Metsämuuronen (2022c,f) included a limited comparison of the behavior of some DCERs in comparison with the traditional counterparts using 1,440 estimates based on real-life datasets. This study is intended to give more systematic information on these new estimators by comparing their characteristics under different conditions.

### Research Questions

Different families of DCERs can be classified by estimators used as the base (e.g., ρ_α_, ρ_*TH*_,ρ_ω_, and ρ_*MAX*_, discussed above), by the score variables (e.g., θ_*X*_, θ_*PC*_, θ_*FA*_, θ_*IRT*_, and θ_*Non*−*Linear*_, discussed below), and by the weighting factors between the item and the score variable (e.g., *R*_*PC*_, *R*_*REG*_, *G, D*, *G*_2_, *D*_2_, *R*_*AC*_, and *E*_*AC*_, discussed below). Combinations are, therefore, many. Systematic studies on the behavior of different combinations would, first, enrich our knowledge of the entire phenomenon and, second, help us to typologize the estimators: which estimators would suit which conditions.

The aim of this study is, first, to compare the characteristics of different DCERs and to form a typology of the estimators: under which conditions which coefficient would be the best option? Second, which combinations of the base and weight factor tend to produce under- or overestimates of reliability in real-life testing settings? In the empirical section, the traditional estimators, alpha, theta, omega, and rho, are used as benchmarks and estimated using their traditional score variables (θ_*X*_, θ_*PC*_, and θ_*FA*_), while DCERs are restricted to the raw score (θ_*X*_).

Before the empirical section, some elementary conceptual points are discussed briefly to make the notation of DCERs understandable. First, the main reason for deflation in reliability, PMC imbedded in the traditional estimators of reliability, is discussed. Second, the traditional model without the elements related to deflation and a general model including these elements are discussed. Finally, different theoretical bases for DCERs related to the forms of ρ_α_, ρ_*TH*_,ρ_ω_, and ρ_*MAX*_ are briefly discussed (for more details, refer to, e.g., Metsämuuronen, 2022a,c).

## Conceptual and Operational Bases For DCERs

### PMC as the Root Cause of Deflation in Reliability

The reason for technical and mechanical deflation in reliability is that traditional estimators of reliability embed PMC in the form of *Rit* and λ_*i*_. PMC is known to be seriously affected by many sources of mechanical error when the scales of two variables are far from each other as is always the case with item and score. In simulations (Metsämuuronen, 2021a, 2022b), seven sources of MEC caused cumulative negative bias in PMC. The sources include extreme item difficulty, a small number of categories in the item, large number of tied cases in the score, and a normally distributed score instead of uniformly distributed. Then, as an example, if the test items are few (leading to a score with a narrow scale with a high number of tied cases), they have an extreme level of difficulty and a binary scale, and the score is normally distributed, we would expect to have radically more deflated item-total correlations leading to radically deflated estimates of reliability, than if the test items are many, they have an average difficulty level, their scale is wide if not continuous, and the score is evenly distributed without tied cases. Notably, this has obvious relevance to the estimates of reliability: If the score does not include tied cases, i.e., because of being continuous or the number of test-takers is small, we expect less deflation in reliability compared with the case that we have a normally distributed or skewed score. However, the effect of skewness in distribution is far less notable than the effect of item difficulty (refer to Metsämuuronen, 2022b, Appendix 1 in Supplementary Material; also, refer later to footnote 4). The issue of the effect of the item distribution is further discussed by Olvera Astivia et al. (2020) and the effect of the scale distribution by Foster (2021) and Xiao and Hau (2022).

Several alternatives for *Rit* and λ_*i*_ are studied from the viewpoint of technical or mechanical errors in the estimates. To some extent, the MEC-affected behavior is known for such traditional estimators of correlation as polychoric correlation coefficient (RPC; Pearson, 1900, 1913; refer to simulations in Metsämuuronen, 2020a,b, 2021a, 2022b), biserial (*R*_*BS*_) and polyserial correlation (*R*_*PS*_) coefficients (Pearson, 1909; see Metsämuuronen, 2020a), r-bireg and r-polyreg correlation (RREG; Livingston and Dorans, 2004; Moses, 2017; refer to Metsämuuronen, 2022b), item–rest correlation (Rir; Henrysson, 1963; refer to Metsämuuronen, 2018, 2021a), lambda and tau (Goodman and Kruskal, 1954; refer to Metsämuuronen, 2020a), coefficient eta (Pearson, 1903, 1905; refer to Metsämuuronen, 2020a, 2022d), delta (D; Somers, 1962; refer to Metsämuuronen, 2020a,b, 2021a,b, 2022b), gamma (G; Goodman and Kruskal, 1954; refer to Metsämuuronen, 2021a,b, 2022b), and tau-a and tau-b (Kendall, 1938, 1948; refer to Metsämuuronen, 2021b, 2022b). Also, some new estimators are developed and studied from this perspective: generalized discrimination index (GDI, Metsämuuronen, 2020c; also refer to the visualization in Metsämuuronen, 2022e) based on Kelley's discrimination index (Kelley, 1939), dimension-corrected *D* (D2; Metsämuuronen, 2020b, 2021a; refer to simulations in Metsämuuronen, 2021a, 2022b), dimension-corrected *G* (G2; Metsämuuronen, 2021a; refer to simulations in Metsämuuronen, 2021a, 2022b), attenuation-corrected *Rit* (*R*_*AC*_; Metsämuuronen, 2022c,d; refer to simulation in Metsämuuronen, 2022b), and attenuation-corrected eta (*E*_*AC*_; Metsämuuronen, 2022d; refer to a simulation in 2022b).

Of the coefficients of correlation, *R*_*PC*_ and *R*_*REG*_ reflect a correlation between unobservable *theoretical* constructs, which may be problematic from the testing theory viewpoint (refer to the critique by Chalmers, 2017); we do not have access to these theoretical constructs. From this viewpoint, such estimators of correlation as *G* and *D* reflect an association between two *observed* constructs; in the settings of measurement modeling, and they strictly indicate the proportion of logically ordered test-takers in a test item after they are ordered by the score (refer to Metsämuuronen, 2021b). For example, if *D* is 0.7, 85% of the observations are logically ordered in the ascending order in the item after they are ordered by the score (*p* = 0.5 × 0.70 + 0.5 = 0.85; refer to Metsämuuronen, 2021b). Because of their conservative nature, with polytomous items having more than three categories, Metsämuuronen (2021a) suggests using *G* and *D* with binary items and with polytomous items having less than four categories. Dimension-corrected *G* and *D* (*G*_2_ and *D*_2_) with semi-trigonometric nature can be used for binary and polytomous items, and in a binary case, *G* = *G*2 and *D* = *D*_2_. Of the attenuation-corrected estimators of correlation (*R*_*AC*_ and *E*_*AC*_), *R*_*AC*_ is more conservative than *E*_*AC*_. This follows strictly from the behavior of *Rit* and coefficient *eta*: except for the binary case, where *Rit* and *eta* give identical estimates, the estimates by *E*_*AC*_ tend to be higher than those by *R*_*AC*_ (refer to Metsämuuronen, 2022d).

The phenomenon of mechanical error in the estimators of correlation is easy to illustrate using two identical (latent) variables with an obvious perfect (latent) correlation (*R* = 1). Let us take the vector of *n* = 1,000 normally distributed cases and double it. Of these identical variables with (obvious) perfect correlation, one (item *g* to be) is divided into four categories [0–3; *df* (*g*) = 3] with difficulty level *p*(*g*) = 0.2 and the other (score *X*) is divided into 61 categories [0–60; *df* (*X*) = 60] with an average difficulty level of *p*(*X*) = 0.5. The difference between the latent correlation and the observed correlation indicates strictly the magnitude of MEC in the estimates (Figure 1). Notably, the estimates by such known estimators of the item–score correlation as *tau-b, Rir, Rit, eta*, and Spearman rank-order correlation cannot reach the latent perfect correlation but, instead, include a remarkable magnitude of deflation (> 0.1 units of correlation) caused by technical and mechanical errors in the estimates. On the contrary, such estimators as *R*_*PC*_, *R*_*REG*_, *G*, *G*_2_, *R*_*AC*_, and *E*_*AC*_ are found MEC-*free* in several conditions (Metsämuuronen, 2022b), and in *D* and *D*_2_, the magnitude of MEC may be nominal depending on the number of tied pairs in the items and score as well as widths of the scales in the items and score (refer to Metsämuuronen, 2021a).

### General Measurement Model Without MEC

Assume a general, simplified, one-latent variable measurement model combining the observed values of an item *g*_*i*_ (*x*_i_), a latent variable (θ), and a weight factor, *w*_*i*_,that links θ with *x*_i_:


(12) $$\begin{array}{l} x_{i}=w_{\text{i}}\theta +e_{i}, \end{array}$$

(e.g., Metsämuuronen, 2022a,c) generalized from the traditional model (e.g., McDonald, 1999; Cheng et al., 2012). In the general model, the theoretical, unobservable θ may be manifested as a varying type of relevantly formed compilation of items including a raw score (θ_*X*_), a principal component score (θ_*PC*_), a factor score (θ_*FA*_), a theta score formed by the item response theory (IRT) or Rasch modeling (θ_*IRT*_), or various non-linear combinations of the items (θ_*Non*−*Linear*_). In the general model, the weight factor *w*_*i*_ is a coefficient of correlation in some form that also includes principal components and factor loadings (λ_*i*_). In all cases, −1 ≤ *w*_*i*_ ≤ +1.

From the coefficient of correlation viewpoint, such estimators as *R*_*PC*_, *R*_*REG*_, *G, D*, *G*_2_, *D*_2_, *R*_*AC*_, and *E*_*AC*_ have been found to be notably better options than PMC (Metsämuuronen, 2022b) as discussed above. In a comparison of eleven sources of MEC, the rough order of the magnitude of MEC (*e*_*wi θ* _*MEC*_; “MEC” in Figure 1) was *e*_*PMCi*θ_*MEC*_ >> *e*_*Di*θ_*MEC*_ >*e*_*D*2*i*θ_*MEC*_ >>*e*_*RREGi*θ_*MEC*_ >*e*_*RPCi*θ_*MEC*_ ≈ *e*_*Gi*θ_*MEC*_ ≈ *e*_*G*2*i*θ_*MEC*_ ≈ *e*_*RACi*θ_*MEC*_ ≈ *e*_*EACi*θ_*MEC*_ ≈ 0 (Metsämuuronen, 2022b). That is, of the better behaving estimators above, on the one hand, *D* is the most conservative option followed by *D*_2_, because both are affected by the number of tied cases in the score variable (refer to Metsämuuronen, 2020b, 2021b). *G* and *D* tend to give obvious underestimates with polytomous items with more than 3–4 categories in the scale, so, *G*_2_ and *D*_2_ are suggested to be used with polytomous items instead of *G* and *D* (Metsämuuronen, 2021a). On the other hand, using *G* and *D* gives quite interesting benchmarking interpretations for the estimates of reliability. Because *G* and *D* strictly indicate the proportion of the logically ordered test-takers in a test item after they are ordered by the score (*p* = 0.5 × *G* + 0.5 and *p* = 0.5 × *D* + 0.5; refer to Metsämuuronen, 2021b), when *D* = 0.8, 90% of the test takers' item responses are in a logical order after the test-takers are ordered by the score. Then, an estimator of reliability using *G* or *D* reflects the proportion of logically ordered test-takers in the entire set of test items.

Notably, the estimates by *eta* and *Rit* are identical with binary items; hence, *R*_*AC*_ and *E*_*AC*_ are identical in binary settings (Metsämuuronen, 2022d). Also, in real-life settings, the sample estimates by *R*_*AC*_ and *E*_*AC*_ tend to mildly overestimate the populations of *R*_*AC*_ and *E*_*AC*_ with polytomous items (Metsämuuronen, 2022c,d). This is caused by the fact that a large population rarely includes deterministic patterns between two variables. Hence, the magnitude of the population values of *R*_*AC*_ and *E*_*AC*_ tend to be somewhat lower than those by sample estimates.

All generally used estimators of correlation give an identical estimate of the correlation for original variables (*g*_*i*_ and θ) and standardized forms of the variables [std(*g*_*i*_) and std(θ)]. Hence, without loss of generality, to lead to a simple form of the estimators of reliability, let us assume that both item (*g*_*i*_) and the manifestation of the latent variable (θ) are standardized, that is, $x_{i},\theta \tilde{\;}\;N\;(0,1)$. Then, the item-wise error variance $\psi_{i}^{2}$ is:


(13) $$\begin{array}{l} \psi_{i}^{2}=1-w_{i}^{2}. \end{array}$$

From eq. (11), the sum of items is:


(14) $$\begin{array}{l} \overset{k}{\underset{i\;=\;1}{\sum }}x_{i}=\overset{k}{\underset{i\;=\;1}{\sum }}w_{i}\theta +\overset{k}{\underset{i\;=\;1}{\sum }}e_{i}, \end{array}$$

where the error variance related to the compilation of the items is:


(15) $$\begin{array}{l} \sigma_{E}^{2}=\overset{k}{\underset{i\;=\;1}{\sum }}\psi_{i}^{2}=\overset{k}{\underset{i\;=\;1}{\sum }}\left(1-w_{i}^{2}\right), \end{array}$$

which can be used in estimating the reliability of the score. If θ is manifested as raw score and *w*_*i*_ as *Rit*, eq. (15) could be used in calculating alpha (Eq. 2), although the practicalities lead to the use of different operationalization of the measurement model. If θ is manifested as a principal component score variable and *w*_*i*_ as principal component loadings, the model in eq. (15) leads to theta (eq. 6). If θ is manifested as a factor score variable and *w*_*i*_ as factor loadings, the model in eq. (15) leads to omega and rho (eqs. 8 and 9, respectively).

### General Measurement Model Including Elements Related to MEC

**Figure 2:**
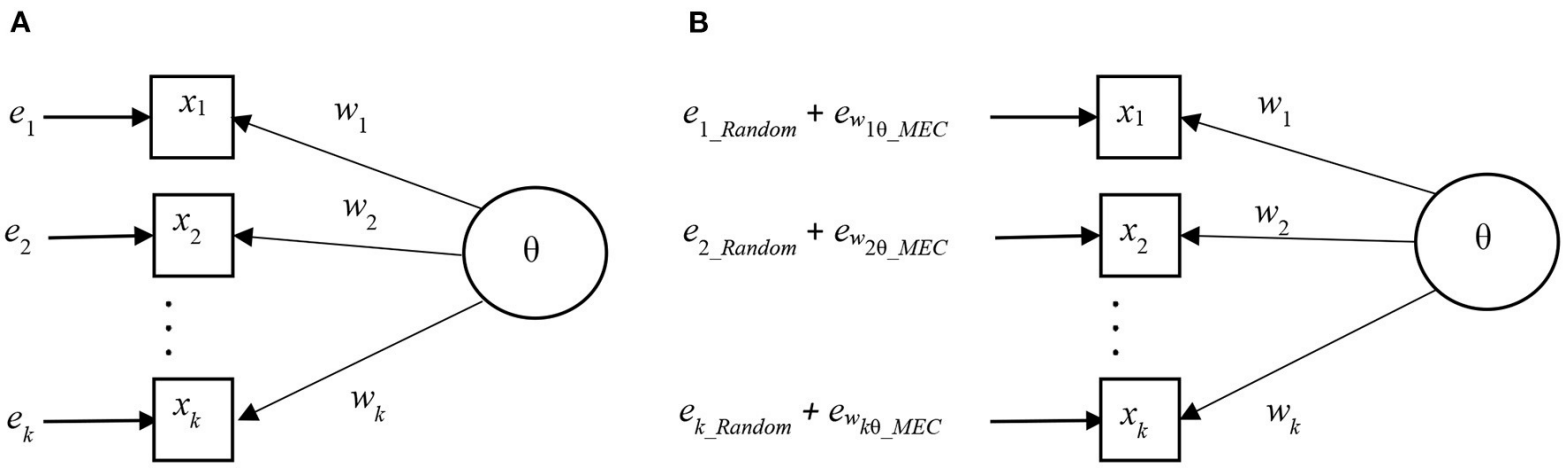
Measurement models without and with elements of MEC. **(A)** Traditional measurement model. **(B)** Measurement model including elements of MEC.

The traditional measurement model related to the estimators of reliability assumes that *Rit* and factor/principal component loadings are deflation-free. This is a too optimistic assumption, as illustrated in Figure 1. Knowing that a certain part of the measurement error is strictly technical or mechanical but that its magnitude could be reduced, Metsämuuronen (2022a,c) suggested reconceptualizing the classic relationship of *X* = *T* + *E*as:


(16) $$\begin{array}{l} X=T+\left(E_{Random}+E_{MEC}\right), \end{array}$$

where the element *E*_*MEC*_ related to deflation is visible. Consequently, we can reconceptualize the measurement model in eq. (12) as:


(17) $$\begin{array}{l} x_{i}=w_{i}\times \theta +\left(e_{i\text{\_}Random}+e_{wi\theta \text{\_}MEC}\right), \end{array}$$

where the element *e*_*wi θ*_*MEC*_ refers to the fact that the magnitude of the mechanical error depends on the characteristics of the weighting factor *w*, item *i*, and score variable θ. In visual forms, the traditional and the MEC-including measurement models are illustrated in Figures 2A,B (Metsämuuronen, 2022a). Notably, in Figure 2, the magnitude of the error in both models is equal, but in Figure 2B, the elements related to MEC are visible.

If we select a weight factor *w*_*i*_ such that the magnitude of the mechanical error is as small as possible, the magnitude of the error component related to deflation may be near zero, that is, *e*_*wi θ* _*MEC*_ ≈ 0. This would lead to an MEC-corrected (MECC) measurement model where the measurement error would be as near the MEC-free condition as possible, that is:


(18) $$\begin{array}{l} x_{i}=w_{i\text{\_}MECC}\times \theta \;+\left(e_{i\text{\_}Random}+e_{wi\theta \text{\_}MEC}\right)\\ \approx w_{i\text{\_}MECC}\times \theta \;+e_{i\text{\_}Random}. \end{array}$$

**Figure 3:**
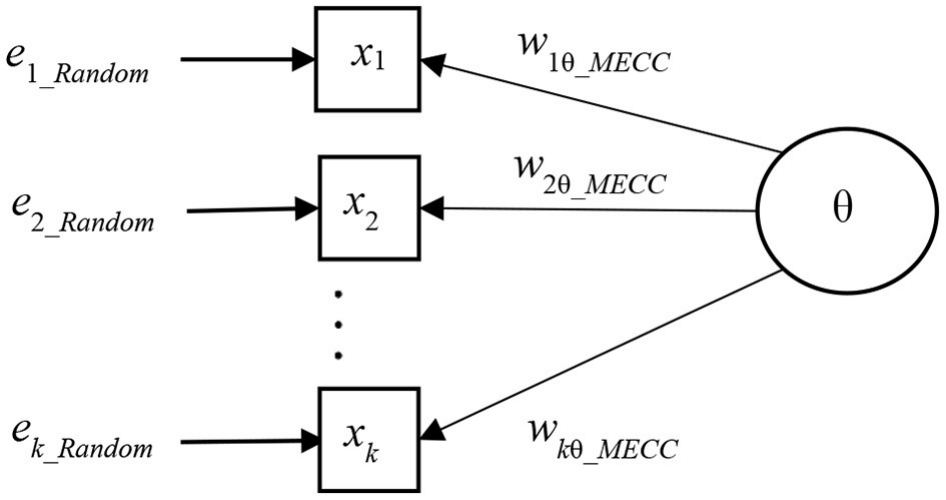
MEC-corrected one-latent variable measurement model.

The measurement model with a near-MEC-free weight factor such as *R*_*PC*_, *R*_*REG*_, *G, D*, *G*_2_, *D*_2_, *R*_*AC*_, and *E*_*AC*_, is illustrated in Figure 3.

This conceptualization leads to item-wise MEC-corrected error variance ($\psi_{i\text{\_}MECC}^{2}$):


(19) $$\begin{array}{l} \sigma_{E\text{\_}MECC}^{2}=\psi_{i\text{\_}MECC}^{2}=1-w_{i\text{\_}MECC}^{2}, \end{array}$$

where $e_{i\text{\_}MECC}\tilde{\;}N\;(0,\psi_{i\text{\_}MECC}^{2})$ and $\psi_{i\text{\_}MECC}^{2}=1-w_{i\text{\_}MECC}^{2}$. Then, after MEC-correction, eq. (15) can be written as:


(20) $$\begin{array}{l} \overset{k}{\underset{i\;=\;1}{\sum }}x_{i}=\overset{k}{\underset{i\;=\;1}{\sum }}w_{i\text{\_}MECC}\times \theta \;+\overset{k}{\underset{i\;=\;1}{\sum }}e_{i\text{\_}Random}, \end{array}$$

and the MEC-corrected error variance of the test score can be written as:


(21) $$\begin{array}{l} \overset{k}{\underset{i\;=\;1}{\sum }}\psi_{i\text{\_}MECC}^{2}=\overset{k}{\underset{i\;=\;1}{\sum }}\left(1-w_{i\text{\_}MECC}^{2}\right), \end{array}$$

This conceptualization leads to short-cuts to estimate deflation-corrected reliability. These estimators are divided into two families as discussed above: on the one hand, *Rit* is replaced by a different coefficient in MECRs: on the other hand, an attenuation-corrected estimator of correlation is used in ACERs. These estimators are short-cuts in the sense that until a sound theoretical basis for a new way of thinking, defining, and estimating reliability is developed, these practical options would lead to a reasonable alternative to deflation-corrected estimates of reliability.

### Theoretical Bases for the Deflation-Corrected Estimators of Reliability

The General (theoretical) bases for different families of DCERs discussed by Metsämuuronen (2022a,c,f) are based on alpha (eq. 3):


(22) $$\begin{array}{l} \rho_{\alpha \text{\_}wi\theta }=\frac{k}{k-1}\left(1-\frac{\overset{k}{\underset{i\;=\;1}{\sum }}\sigma_{i}^{2}}{{\left(\overset{k}{\underset{i\;=\;1}{\sum }}\sigma_{i}w_{i\theta }\right)}^{2}}\right), \end{array}$$

theta (eq. 5):


(23) $$\begin{array}{l} \rho_{TH\text{\_}wi\theta }=\frac{k}{k-1}\left(1-\frac{1}{\overset{k}{\underset{i\;=\;1}{\sum }}w_{i\theta }^{2}}\right), \end{array}$$

omega (eq. 6):


(24) $$\begin{array}{l} \rho_{\omega \text{\_}wi\theta }=\frac{{\left(\overset{k}{\underset{i\;=\;1}{\sum }}w_{i\theta }\right)}^{2}}{{\left(\overset{k}{\underset{i\;=\;1}{\sum }}w_{i\theta }\right)}^{2}+\overset{k}{\underset{g\;=\;1}{\sum }}\left(1-w_{i\theta }^{2}\right)}, \end{array}$$

or rho (eq. 7):


(25) $$\begin{array}{l} \rho_{MAX\text{\_}wi\theta }=\frac{1}{1+\frac{1}{\overset{k}{\underset{i\;=\;1}{\sum }}\left(w_{i\theta }^{2}/\left(1-w_{i\theta }^{2}\right)\right)}}, \end{array}$$

where the notation *w*_*i* θ_ refers to the fact that the magnitude of the estimate depends on three things: characteristics of the weight factor (*w*), the item (*i*), and the score variable (θ) as a manifestation of the latent trait as discussed above. Other bases could also be used. However, using theta, omega, and rho outside of their traditional context is debatable. Here, it is assumed that the estimators *could* be used as independent estimators; this seems consistent with the general measurement model discussed above. Alternatively, we may think that the estimates we get using *R*_*PC*_, *R*_*REG*_, *G, D*, *G*_2_, *D*_2_, *R*_*AC*_, or *E*_*AC*_ instead of the traditional λ_*i*_ are outcomes of renewed procedures on principal component and factor analysis where the factor loadings are, i.e., *R*_*PC*_ and *G*_2_ instead of PMC (cl. ordinal theta by Zumbo et al., 2007).

The practical characteristics of the estimators are studied in the empirical section. From a theoretical viewpoint, in hypothetic extreme datasets with deterministic item discrimination in *all* items leading to *R*_*PCi*_ =*R*_*PCj*_ ≈ *G*_*i*_ =*G*_*j*_ = *G*_2i_ = *G*_2j_ = *R*_*ACi*_ = *R*_*ACj*_ = *E*_*ACi*_ = *E*_*ACj*_ ≡ 1,^2^ estimators based on rho (eq. 25) could not be used, because this would require division by zero, which is not defined. However, DCERs based on theta and omega (eqs. 23 and 24) would lead to perfect reliability (*REL* = 1):


(26) $$\begin{array}{l} \rho_{TH\text{\_}RPCi\theta }^{Max}\approx \rho_{TH\text{\_}Gi\theta }^{Max}=\rho_{TH\text{\_}RACi\theta }^{Max}\\ \;=k/\left(k-1\right)\left(1-1/k\right)\equiv 1 \end{array}$$

and


(27) $$\begin{array}{l} \rho_{\omega \text{\_}RPCi\theta }^{Max}\approx \rho_{\omega \text{\_}Gi\theta }^{Max}=\rho_{\omega \text{\_}RACi\theta }^{Max}={\left(k\right)}^{2}/\left({\left(k\right)}^{2}+0\right)\equiv 1. \end{array}$$

The maximum value by the estimators based on alpha (eq. 22) is:


(28) $$\begin{array}{l} \rho_{\alpha \text{\_}RPCi\theta }^{Max}\approx \rho_{\alpha \text{\_}Gi\theta }^{Max}=\rho_{\alpha \text{\_}RACi\theta }^{Max}\\ \;=\frac{k}{k-1}\left(1-\overset{k}{\underset{i\;=\;1}{\sum }}\sigma_{i}^{2}/{\left(\overset{k}{\underset{i\;=\;1}{\sum }}\sigma_{i}\right)}^{2}\right). \end{array}$$

Hence, estimators based on alpha can reach the value $\rho_{\alpha \text{\_}RPCi\;\theta \;}^{Max}\approx \rho_{\alpha \text{\_}Gi\;\theta \;}^{Max}=\rho_{\alpha \text{\_}RACi\;\theta \;}^{Max}=1$ only when all item variances are equal (σ_*i*_ = σ_*j*_ = σ), that is, for instance, when the items are standardized. In the case


(29) $$\begin{array}{l} {\rho_{\alpha \text{\_}}}_{RPCi\theta }\approx {\rho_{\alpha \text{\_}}}_{Gi\theta }={\rho_{\alpha \text{\_}}}_{Di\theta }\\ \;=k/\left(k-1\right)\left(1-k\sigma^{2}/{\left(k\sigma \right)}^{2}\right)\\ \;=k/\left(k-1\right)\left(1-1/k\right)\equiv 1 \end{array}$$

Notably, in the theoretical case, all the item–score correlations are equal to 0, and except for those based on omega, none of the estimators are defined. This is inherited from the original estimators: those that are not defined when all correlations or loadings are 0.

## Methodology

### Measurement Model and Estimators Used in the Empirical Section

In the empirical section, the characteristics of different types of DCERs are compared by varying the characteristics of *w* and *i* in a real-life setting with finite or small sample sizes. The general measurement model discussed above is applied in the empirical section. Formulae (22) to (25) are used as bases for the estimators. The raw score (θ_*X*_) is used as the manifestation of θ and *R*_*PC*_, *R*_*REG*_, *G, D*, *G*_2_, *D*_2_, *R*_*AC*_, and *E*_*AC*_ as weight factors. The estimators of correlation and their estimation are described in Appendix 1 in Supplementary Material (refer to details in, e.g., Metsämuuronen, 2022b). The estimates by the traditional estimators ρ_α_, ρ_TH_, ρ_ω_, and ρ_MAX_ (eqs. 3, 6, 8, and 9), with their traditional original score variables (θ_*X*_ for alpha, θ_PC_ for theta, and θ_FA_ with ML estimation for omega and rho) and weight factor (*Rit* for alpha and λ_*i*_ for theta, omega, and rho), are used as benchmarks to the DCERs. With only two items with a wide-scale, principal axis factoring (PAF), instead of ML, is conducted to estimate the factor loadings.

In the empirical section, the estimators are named based on eqs. (22) to (25). For example, ρ_*MAX*_*RPCiX*_ refers to eq. (25) where the base is the formula of rho (ρ_*MAX*_), the weight factor is *R*_*PC*_, and the score variable is the raw score (θ_*X*_). In the Figures and Tables, this is expressed as “RhoRPC.” Similarly, the traditional estimators are referred to as “AlphaRit,” “ThetaPC,” “OmegaML,” and “RhoML” or by an attribute “traditional” such as “Alpha traditional.”

The estimators and estimates are also compared from the viewpoint of their capability of reflecting the population value. A simple statistic for this is used: the difference between the sample estimate and the population value (*d*). When *d* > 0, the true correlation is overestimated, and when *d* < 0, the sample estimate underestimates the population estimate. In the Figures and Tables, this difference related to a specific estimator is referred to as “dRhoRPC” and “dRho traditional”.

### Datasets Used and Tests Conducted in the Study

A real-world dataset of 4,022 nationally represented test-takers of a mathematics test with 30 binary items (FINEEC, 2018) is used as the “population”. In the original dataset, ρ_α_ = 0.885, ρ_*TH*_ = 0.89, ρ_ω_ = 0.887, and ρ_*MAX*_ = 0.895; the difficulty levels of the items ranged 0.24 < *p* < 0.95, with the average $\bar{p}$ = 0.63; and item discrimination ranged 0.332 < *Rit* < 0.627 with the average $\bar{Rit}=\;0.481$.

Ten random samples with *n* = 25, 50, 100, and 200 test-takers were picked from the original dataset. These finite samples imitate different sizes of real-world sample sizes, ranging from a test for a large student group (*n* = 200) to classroom testing (*n* = 25). In each of the 10 × 4 datasets, 36 tests were produced by varying the number and difficulty levels of the items and the length of the scale of the score [*df* (*X*) = number of categories in the scale−1] and the item [*df* (*g*) = number of categories in the scale−1]. The polytomous items were constructed as sums of the original binary items. Thus, the datasets^3^ consists of 14,880 partly related test items from 1,440 partly related tests with a varying number of test items (*k* = 2–30, $\bar{k}=10.33$, SD 8.621) and test-takers (*n* = 25, 50, 100, and 200), number of categories in the items [*df* (*g*) = 1–14, $\bar{df\left(g\right)}=4.57$, SD 3.480], and in the score [*df* (*X*) = 10–27, $\bar{df\left(X\right)}=18.06$, SD 3.908], the average difficulty levels ($\bar{p}$= 0.50–0.76, $\bar{\bar{p}}$= 0.66. SD 0.052), and the lower bound of reliabilities (ρ_α_ = 0.55–0.93, ${\overset{-}{\rho }}_{\alpha }=0.850$, SD 0.049).

## Results

Because previous studies related to DCERs have been fragmented, this study intends to offer a more systematic comparison of the estimators with a larger number of estimates. In doing so, five characteristics of DCERs are studied: their general tendencies in comparison with traditional estimators, their capability to reflect the population value, the effect of the sample size in the estimators, the effect of the number of categories in the score, and the effect of test difficulty. In what follows, mainly DCERs based on the form of omega (“deflation-corrected omega”) are presented in the text, and all estimators in the comparison are collected in Appendix 2 in Supplementary Material.

### General Tendencies of DCERs

**Figure 4:**
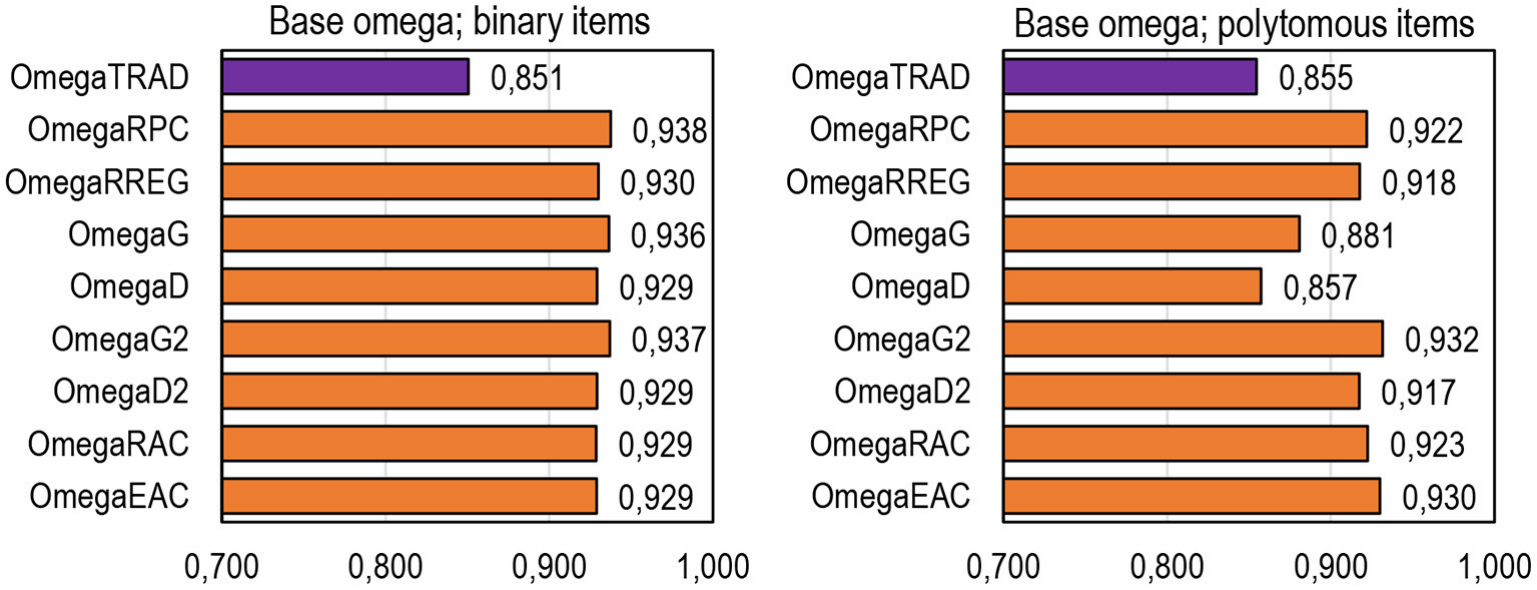
Average estimates by DCERs based on the form of omega.

Of the general tendencies of DCERs, three are highlighted. First, in comparison with the traditional estimators based on *Rit* and λ_*i*_, all DCERs in the simulation give, in general, higher estimates. This is specifically true with binary datasets where all DCERs give systematically and consistently almost the same estimate, which is 0.07–0.09 units higher than the traditional estimates (Table 1; Figure 4; refer also to Appendix 2 in Supplementary Material). With binary items, all DCERs, irrespective of the base, suggest that the reliability of the (original) test would rather be 0.91–0.94 and not 0.85–0.88 as suggested by the traditional estimators. This higher magnitude of the estimates is caused by the less-deflated estimates of correlation with items of extreme difficulty level by the alternative estimators in comparison with PMC. Although the true reliability of the original real-life dataset is unknown, the unified voice of DCERs speaks of the possibility that they reflect the *same* (latent) true reliability. Notably, the differences between traditional estimates and those by DCERs are remarkably smaller than the ones in examples described by Gadermann et al. (2012) and Metsämuuronen (2022a,c), and in extreme cases, the difference is reported to be 0.4–0.6 units of reliability. The smaller difference is caused by the fact that the datasets used in the simulation do not include extremely easy or extremely difficult items or tests.

**Table 1:** Average estimates of reliability and deviance from the population value in simulation.

	**Traditionalestimators**				**MCER (R**_**PC**_ **)**				**MCER (R**_**REG**_ **)**			
**Base**	**Alpha**	**Theta**	**Omega**	**Rho**	**Alpha**	**Theta**	**Omega**	**Rho**	**Alpha**	**Theta**	**Omega**	**Rho**
Estimate^a^	0.850	0.858	0.854	0.875	0.891	0.896	0.925	0.935	0.885	0.890	0.920	0.928
Deviation^b^	−0,016	−0,001	−0,012	0,012	−0,009	−0,002	−0,005	0,008	−0,005	0,001	−0,001	0,007
*N*	1,440	1,440	1,394	1,384	1,440	1,440	1,440	1,418	1,440	1,440	1,440	1,421
	**MCER (G)**				**MCER (D)**				**MCER (G**_**2**_ **)**			
**Base**	**Alpha**	**Theta**	**Omega**	**Rho**	**Alpha**	**Theta**	**Omega**	**Rho**	**Alpha**	**Theta**	**Omega**	**Rho**
Estimate^a^	0.831	0.834	0.893	0.904	0.789	0.796	0.873	0.883	0.905	0.910	0.933	0.942
Deviation^b^	−0,009	−0,005	−0,005	0,009	−0,010	−0,002	−0,005	0,009	−0,009	−0,001	−0,005	0,009
*N*	1,440	1,440	1,440	1,418	1,440	1,440	1,440	1,426	1,440	1,440	1,440	1,418
	**MCER (D**_**2**_ **)**				**ACER (R**_**AC**_ **)**				**ACER (E**_**AC**_ **)**			
**Base**	**Alpha**	**Theta**	**Omega**	**Rho**	**Alpha**	**Theta**	**Omega**	**Rho**	**Alpha**	**Theta**	**Omega**	**Rho**
Estimate^a^	0.884	0.890	0.920	0.930	0.891	0.897	0.924	0.934	0.901	0.906	0.930	0.939
Deviation^b^	−0,010	−0,002	−0,005	0,009	−0,007	0,001	−0,003	0,010	−0,006	0,001	−0,002	0,010
*N*	1,440	1,440	1,440	1,426	1,440	1,440	1,440	1,418	1,440	1,440	1,440	1,418

a *Average estimate*.

b *Average deviation between the sample and population estimates*.

Second, when the number of categories in the items exceeds 4, *G* and *D* tend to give an obvious underestimation of the item–score association (refer to, e.g., Metsämuuronen, 2021a). Hence, we obtain notably low estimates of reliability using alpha and theta as bases for the DCERs with items that have a wide scale (refer to Figure 4; Appendix 2 in Supplementary Material). In these cases, using the dimension-corrected estimators *G*_2_ and *D*_2_ would be better, with binary items *G* = *G*_2_ and *D* = *D*_2_. Using *G*_2_ and *D*_2_ as the linking factor with polytomous items seems to give largely the same magnitude of reliability as given by *R*_*PC*_ and *R*_*REG*_.

Third, using rho as the base may lead to missing estimates, specifically with small sample sizes. Datasets with the smallest sample size in the simulation produce a remarkable number of deterministic patterns (6% of the estimates with *n* = 25) where the estimates based on rho are not defined. Then, factually, the number of estimates is 1,418 (instead of 1,440) for estimators based on rho (refer to Table 1). Small sample sizes are prone to produce not only deterministic patterns where rho cannot be calculated at all but also near-deterministic patterns leading to (artificially) high estimates. This characteristic seems to be inherited also to DCERs based on rho: the estimates based on rho with binary items (0.94–0.96) are suspiciously high in comparison with the estimators based on theta and omega (0.93–0.94; refer to Appendix 2 in Supplementary Material). This is related to the note by Aquirre-Urreta et al. (2019) that traditional rho tends to give overestimates with finite samples.

### The Capability of DCERs to Reflect Population Reliability

**Figure 5:**
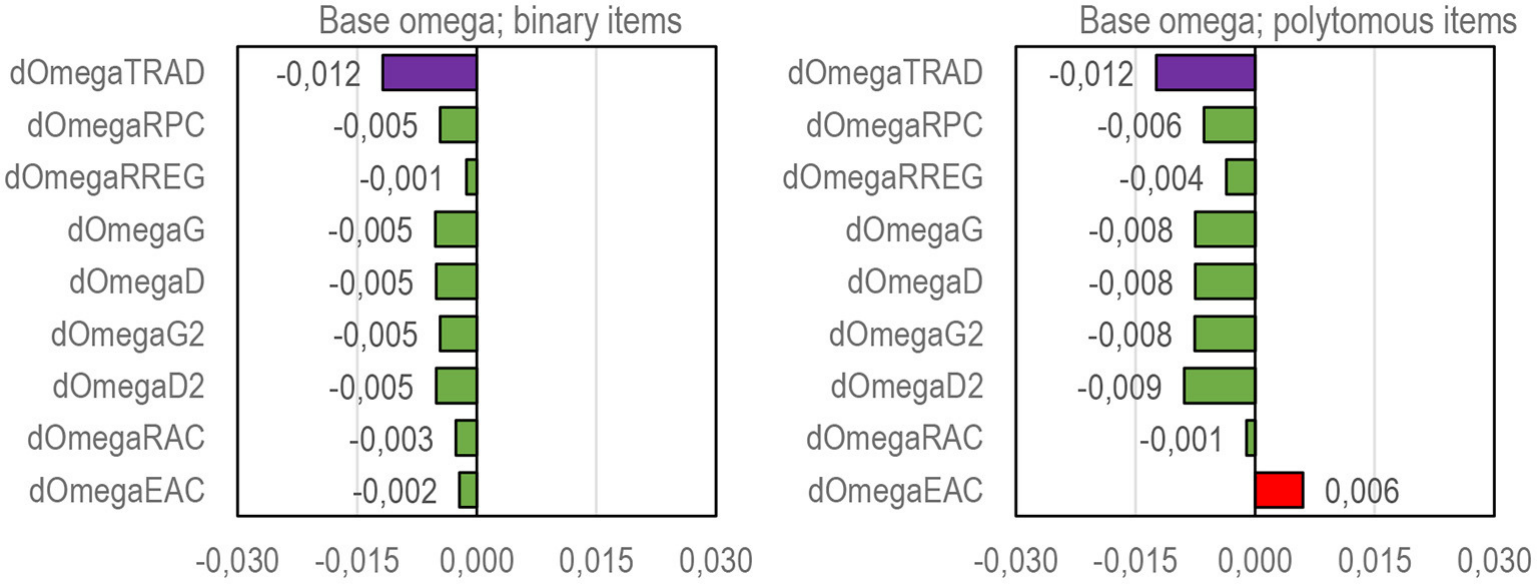
Deviance between sample and population estimates by DCERs based on the form of omega.

Another aspect of the general tendencies is how well sample estimates reflect population estimates. This is illustrated in Figure 5 and Appendix 2 in Supplementary Material, and four points are highlighted here. First, DCERs based on alpha, theta, and omega are conservative: they tend to produce estimates where the magnitude is lower than population reliability. In contrast, DCERs based on rho tend to be liberal: the estimates tend to overestimate population reliability, especially with binary items (refer to Appendix 2 in Supplementary Material). Second, sample estimators using *E*_*AC*_ as a linking factor tend to overestimate population reliability based on *E*_*AC*_. Notably, the factual estimates of reliability seem not to be overestimated when *E*_*AC*_ is used (refer to Figure 4 above). Third, estimators based on the form of theta and rho tend to be more stable than those using alpha and omega, theta in binary settings, and rho with polytomous settings (except when *R*_*AC*_ or *E*_*AC*_ are used as the linking factor; refer to Appendix 2 in Supplementary Material). In estimators based on theta and rho, the deviance between the sample and population estimates is generally around 0.001–0.002 units of reliability. With estimators based on alpha and omega, the deviance is around 0.01–0.02 units of reliability.

**Figure 6:**
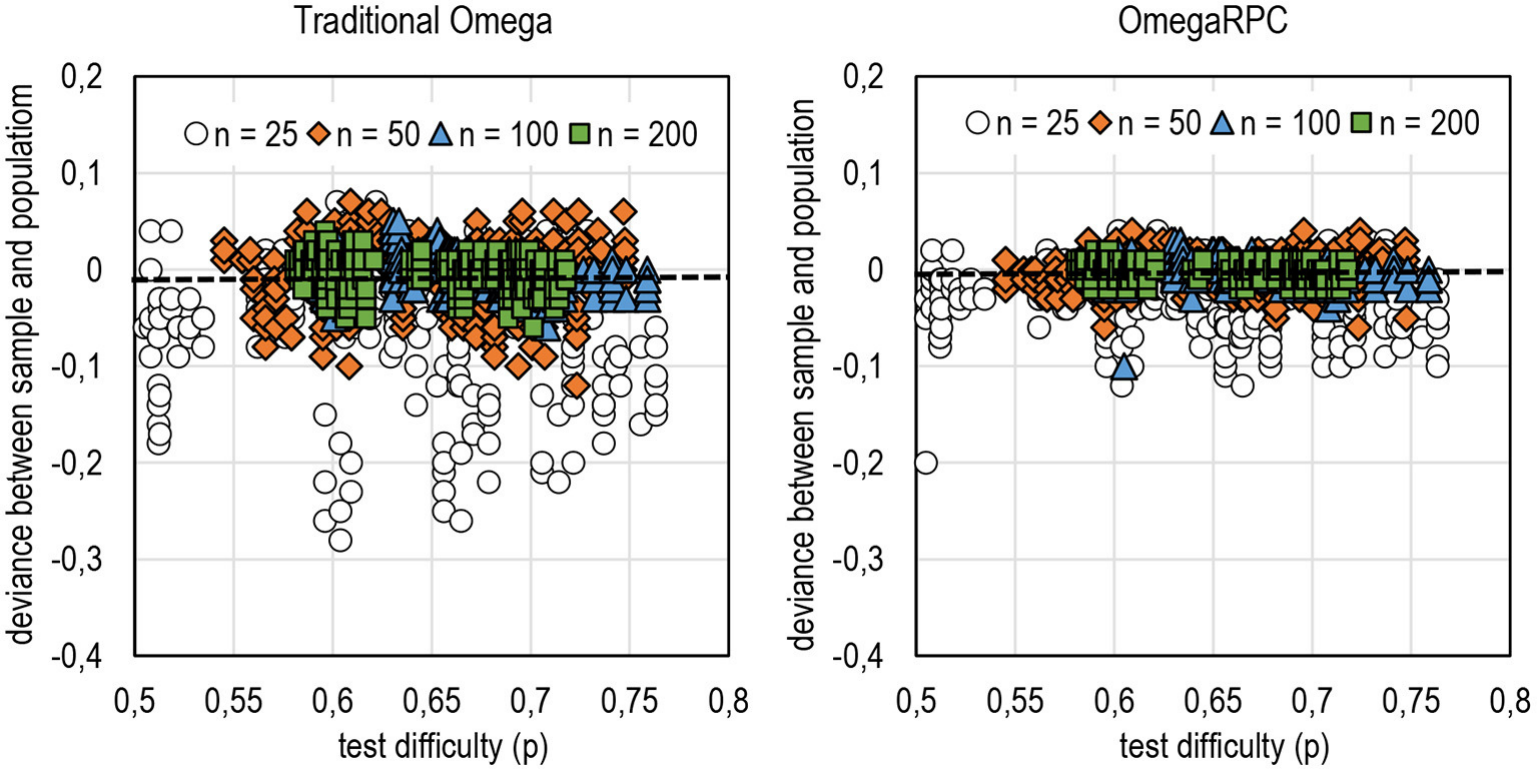
Deviance between sample and population estimates by a DCER based on omega.

Fourth, although the general tendencies show only mild deviance between sample and population, single estimates in the sample may be far off the population value. Figure 6 illustrates how widely the estimates may deviate from the population values, specifically with small sample sizes. The reason for the wide deviance with small sample sizes, specifically when using the traditional omega, is that even one test-taker may have a notable effect on changing the correlations between the item and score and, in some cases, even from positive (in the population) to negative in the sample (refer to examples in Metsämuuronen, 2022b).

**Figure 7:**
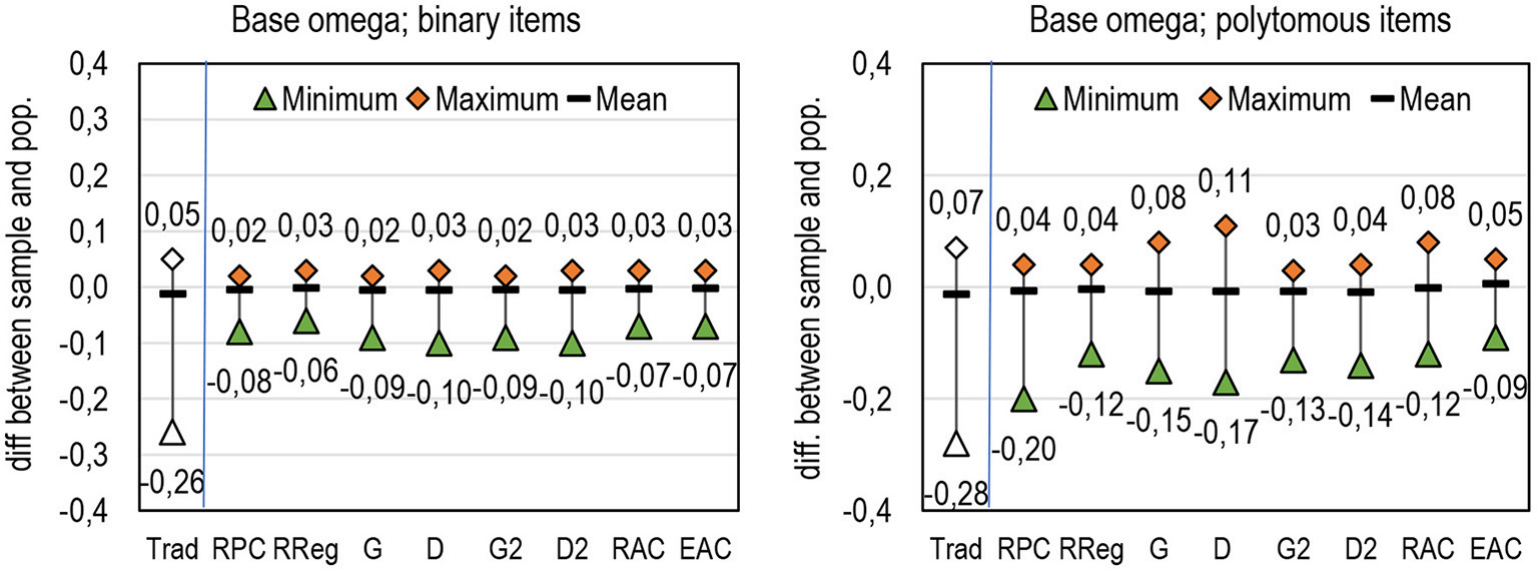
Deviance between sample and population estimates in DCERs based on omega.

Generally, except with estimators based on alpha, the deviance between the sample and population estimates seems notably smaller by DCERs than by traditional estimators (refer to Figure 7; Appendix 2 in Supplementary Material). Specifically, this is true with binary items. The traditional theta seems to give relatively more stable estimates even without correction for deflation. Notably, the wide range in deviance between the sample and population estimates with polytomous items when *G* or *D* are used as the linking factor and alpha as the base is caused by the fact that *G* and *D* tend to give obvious underestimation when the number of categories in item exceeds 3–4.

### Effect of Sample Size on DCERs

**Figure 8:**
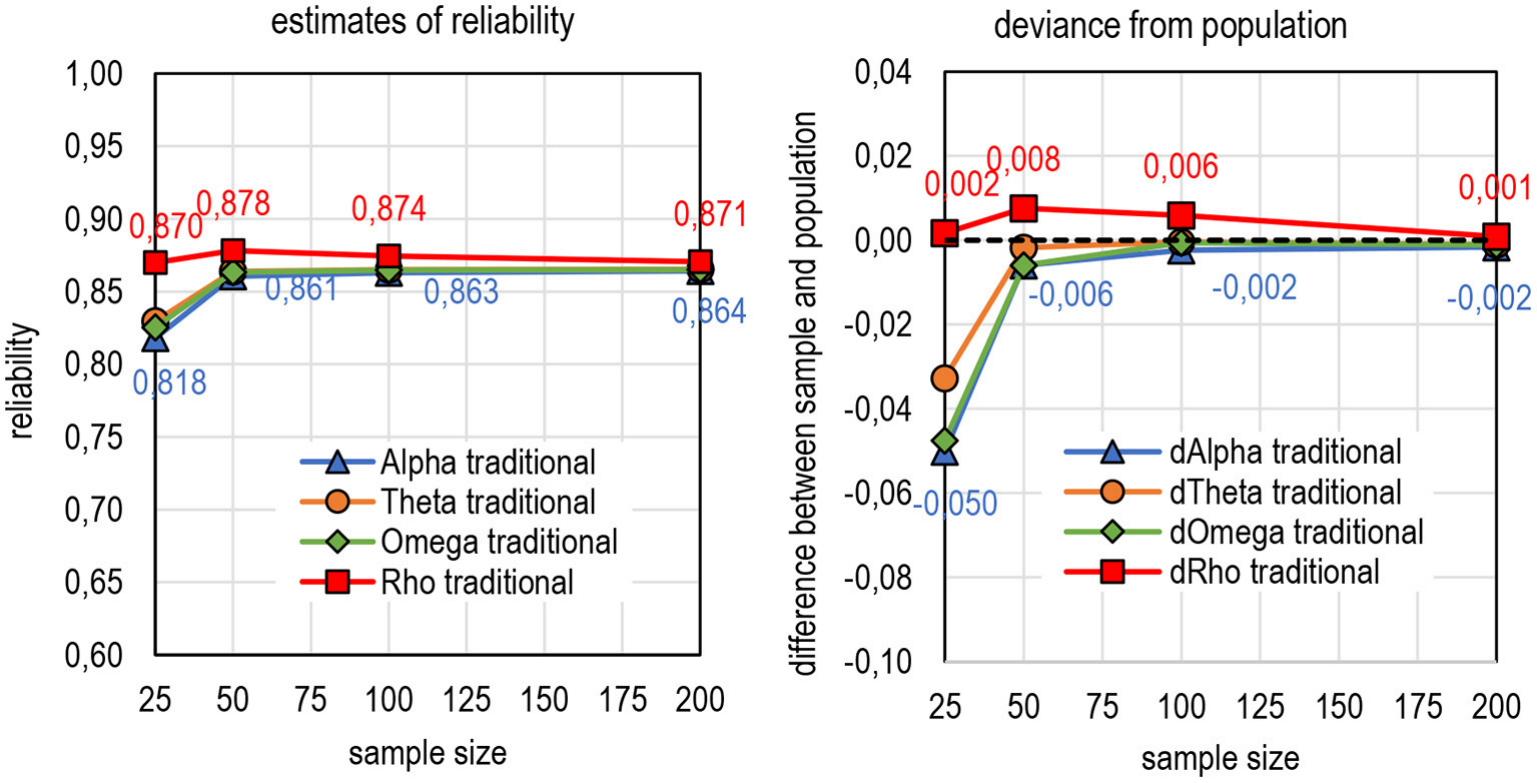
Average estimates of reliability and deviance from the population by sample size.

**Figure 9:**
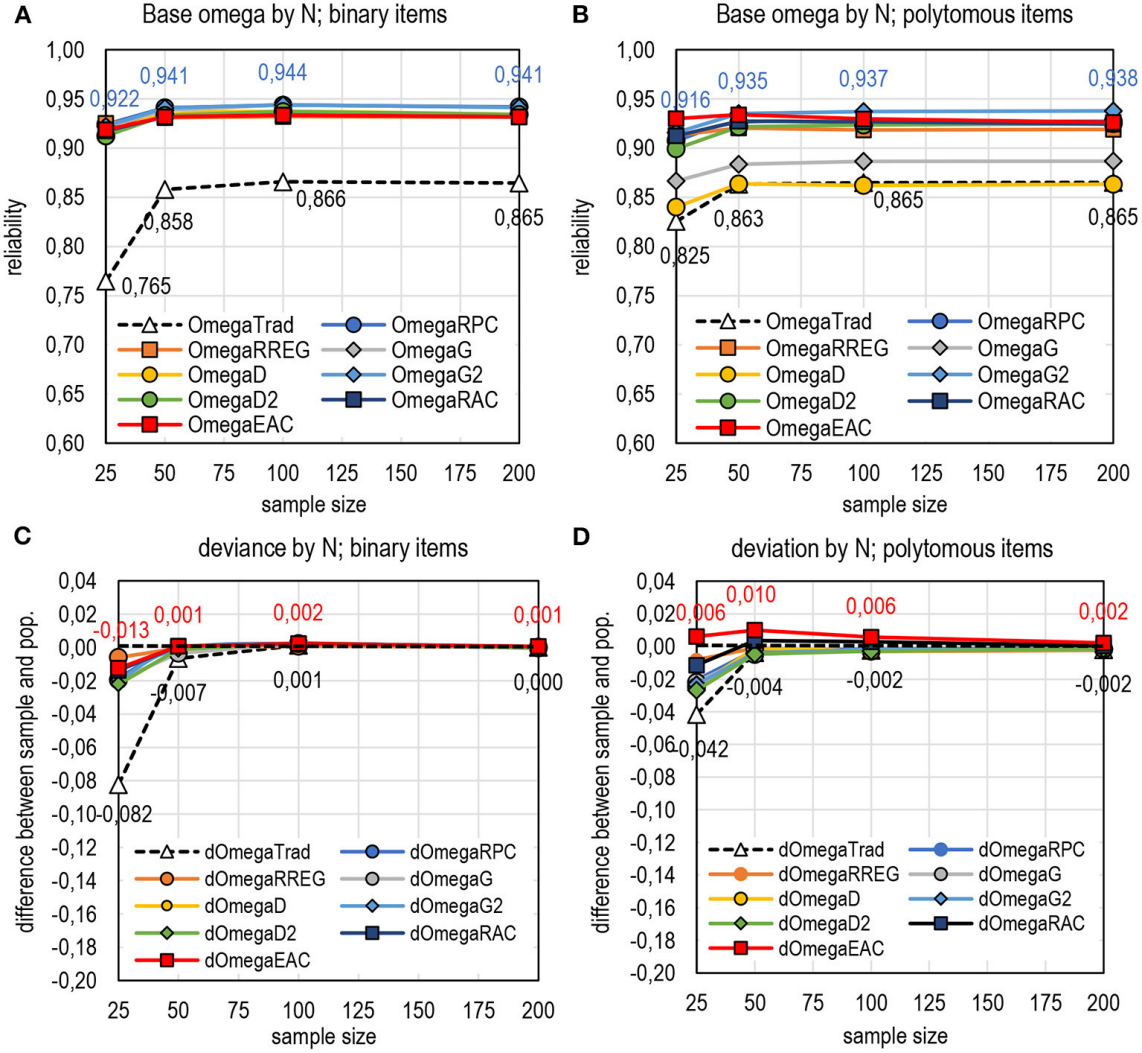
The behavior of DCERs based on omega by sample size. **(A)** Base omega by N; binary items. **(B)** Base omega by N; polytomous items. **(C)** Deviance by N; binary items. **(D)** Deviation by N; polytomous items.

As a benchmark to DCERs in Figure 9, Figure 8 illustrates the behavior of the traditional estimators by sample size (refer to details in Appendix 2 in Supplementary Material). All the conservative estimators (alpha, theta, and omega) tend to give estimates that deviate notably from the population value when the sample size is very small (*n* = 25). When the sample size reaches *n* = 50, the estimates are relatively stable. Theta seems to be the most stable when it comes to reflecting the population value. The estimates by rho are higher than others, but it also tends to overestimate mildly population reliability (up to 0.008 units of reliability) with small sample sizes.

The estimates by DCERs differ notably depending on whether binary or polytomous items are used. With binary items, all DCERs give largely the same estimates, while with polytomous items, DCERs using *G* and *D* as the linking factor underestimate reliability irrespective of the sample size (refer to Figure 9 and more details in Appendix 2 in Supplementary Material). In both cases, the estimates are stable when the sample size is *n* = 50 or higher. All the estimators underestimate population reliability with a very small sample size (*n* = 25).

It seems that DCERs give a notable advantage when the sample size is small. This is true specifically with binary items; the estimates by DCERs tend to be closer to the population value in comparison with the traditional estimators. Omega would benefit the most by changing the linking factor. With polytomous items, DCERs using *E*_*AC*_ as the linking factor tend to overestimate the population value, although the factual estimates do not exceed the magnitude of the estimates using *G*_2_ as the linking factor.

Traditional alpha, omega, and rho seem to benefit if the linking factor is changed from PMC to any of the item–score correlations used for comparison. The estimators using bi- and polyreg correlation coefficient (*R*_*REG*_) with very small sample sizes seem to give more stable estimates than other estimators of correlation, and the estimates based on theta seem to be relatively stable even with small sample sizes and without changing the linking factor.

### Effect of Number of Categories in the Score on DCERs

**Figure 10:**
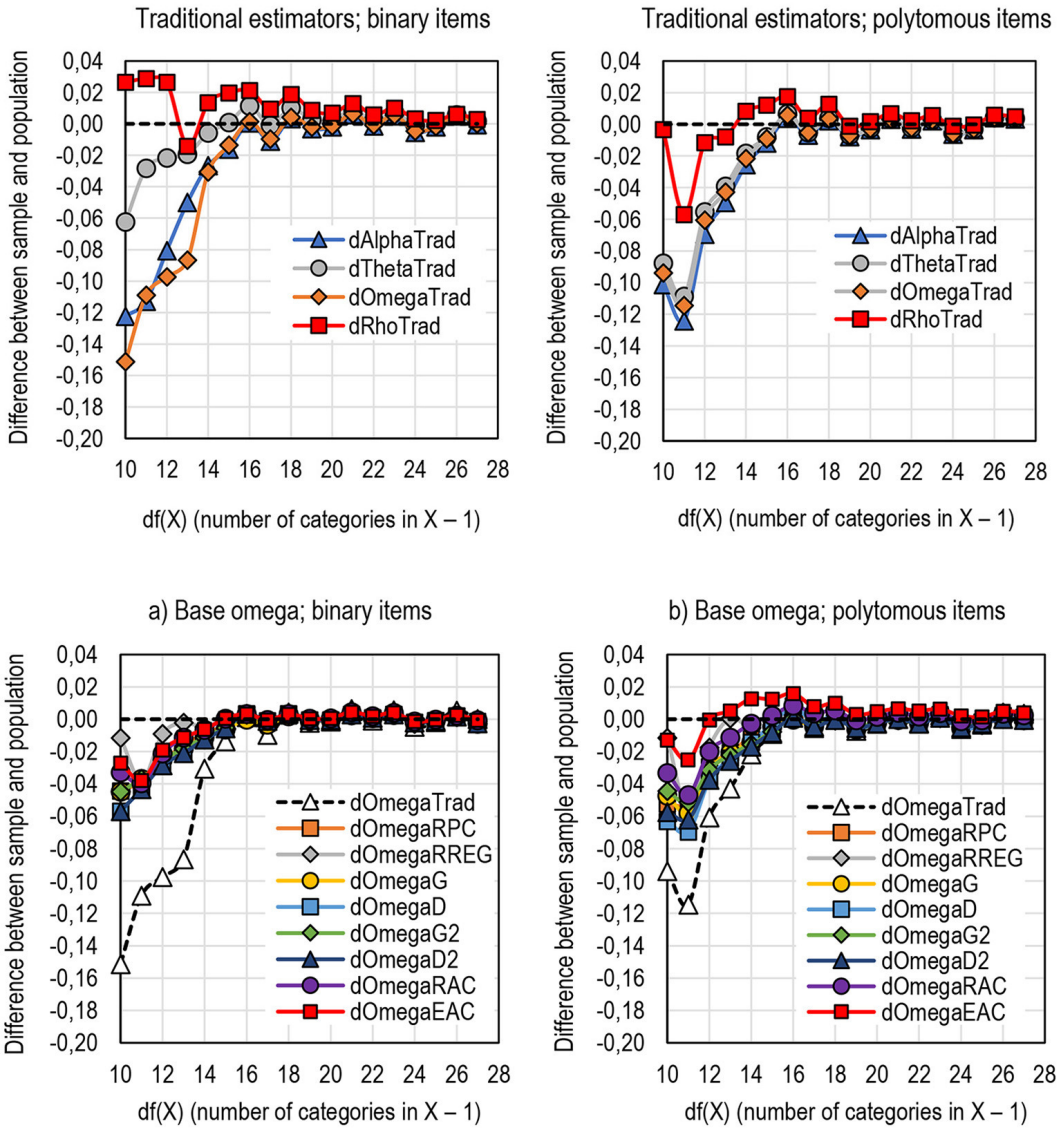
The behavior of traditional estimators of reliability by the width of the score [df(X)]. The behavior of DCERs by the width of the score [df(X)]. **(a)** Base omega; binary items. **(b)** Base omega; polytomous items.

The dataset used in simulation is limited when it comes to the number of categories in the score variable. Because of the limitations in the original dataset, only scores with a number of categories ranging from 11 to 31 [*df* (*X*) = 10–30] could be used. However, it seems that all the estimators give stable estimates when the number of categories in the score exceeds 20 (Figures 10a,b).

Among the traditional estimators, alpha and omega seem quite unstable when the scale of the score is narrow [*df* (*X*) < 15], and the reliability of the population may be underestimated by more than 0.1 units (Figure 10b). From this viewpoint, the estimates by theta are notably closer to the population values as the reliability is underestimated by less than 0.06 units with binary items. The estimates by rho tends to overestimate reliability by up to 0.03 units with scores with a narrow scale, although the estimates tend to be rather stable with polytomous items even when the score has a narrow scale.

When it comes to DCERs, in general, those using a conservative base (alpha, theta, and omega) tend to underestimate population reliability less than the traditional estimators, specifically with scores with a narrow scale [*df* (*X*) < 15] and binary items, whereas those based on a liberal base (rho), tend to less overestimate population reliability than traditional estimators with short tests (Figure 10b; Appendix 2 in Supplementary Material). Although the DCERs that use *E*_*AC*_ as the linking factor tend to overestimate reliability with polytomous items (refer to above), the estimates tend to be closest to the population value with polytomous items and very short tests [df(X) < 14].

### Effect of Test Difficulty on DCERs

**Figure 11:**
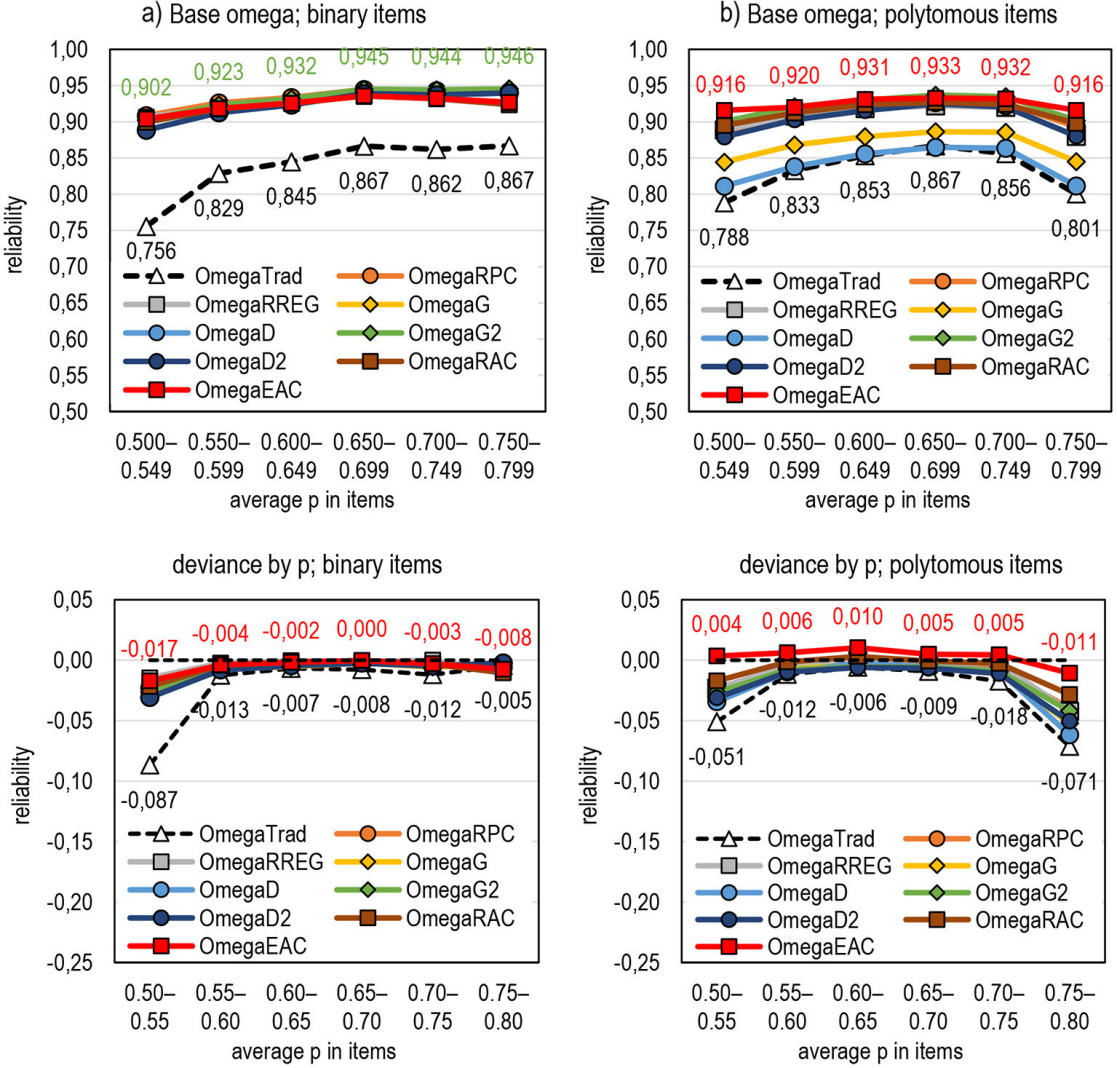
The behavior of DCERs by test difficulty (the highest and traditional estimates are highlighted). **(a)** Base omega; binary items. **(b)** Base omega; polytomous items.

Lastly, the estimators are compared by their behavior for tests with different difficulty levels. Notably, the dataset used in the simulation does not allow comparing them with extremely difficult or extremely easy tests; in such tests, *Rit* is the most vulnerable. Still, some comparisons are conducted although the number of “difficult” (average proportion of correct answers in the items is $\bar{p}$ < 0.55) and “easy” tests ($\bar{p}$> 0.75) is small. Figures 11a,b (refer also to Appendix 2 in Supplementary Material) illustrate the behavior of omega and the related DCERs regarding test difficulty, and three points are highlighted.

First, of the traditional estimators, alpha and omega tend to be more affected by test difficulty than theta and rho. Alpha and omega tend to underestimate reliability in both extremes. Theta seems relatively stable with binary items but is affected by test difficulty with polytomous items. Rho is stable, although it seems to overestimate reliability irrespective of test difficulty if the difficulty level is not extreme.

Second, with binary items, the magnitude of the estimates by DCERs tends to be notably higher and more stable than by the traditional estimators irrespective of test difficulty. A specific advantage of DCERs is with a test of extreme difficulty level where the traditional estimators tend to give lower values. This is specifically true with estimators based on alpha and omega; it seems that the traditional alpha and omega would benefit most by changing the linking factor.

Third, with polytomous items, using *R*_*AC*_ or *E*_*AC*_ as a linking factor seems to produce the most stable estimates irrespective of the base used and test difficulty. *E*_*AC*_ tends to overestimate reliability mildly, but the factual estimates tend not to differ from those where *G*_2_ is used. Except for the estimators that use *D* and *G*, the differences between the estimates are small.

## Conclusions, Discussion, and Restrictions

### Results in a Nutshell

The starting point of this article was two-fold. First, the empirical findings indicate that the estimates by the traditional estimators of reliability such as alpha, theta, omega, and rho tend to be deflated, and the magnitude of deflation may be remarkable with certain types of datasets, typically with tests including items of extreme difficulty level. Second, the main reason for the deflation in the estimates of reliability is the mechanical error related to estimates of the item–score correlation embedded in the widely used traditional estimators of reliability. The behavior of alternative estimators for *Rit* has been studied, and short-cut estimators of reliability that produce deflation-corrected estimates have been proposed based on replacing *Rit* with an alternative, which gives a radically smaller magnitude of deflation. Some of these alternatives are *R*_*PC*_, *R*_*REG*_, *G, D*, *G*_2_, *D*_2_, *R*_*AC*_, and *E*_*AC*_, which are discussed in the empirical section.

Different families of DCERs can be classified by the estimator used as the base, by score variables, and by weighting factors between item and score variable. Studies concerning DCERs have been either at a very initial stage, they have offered just some examples of the new possibility, they have been based on small datasets and have been fragmentary, or the simulations have made only a limited comparison of the behavior of some DCERs with their traditional counterparts. The aim of this study was to conduct a more systematic comparison of the behavior of different combinations of these elements and to typologize estimators that would show which estimator suits which situations. The simulation used here was based on finite sample sizes relevant to many real-life testing settings (*n* ≤ 200). Although the simulation conducted and the dataset used have their restrictions, which will be discussed later, seven main outcomes may be presented here:

1. Regardless of the base and linking factor used, DCERs tend to give higher estimates than traditional estimators. This is because of higher magnitudes of the item–score correlations obtained by the alternative estimators than by the traditional *Rit*.
2. Not only are their estimates higher, DCERs seems to tend to produce estimates that are closer to the population value than the traditional estimators do.
3. Although the true reliability of the original real-life dataset is unknown, the unified voice of the DCERs, specifically with binary items, speaks that they reflect the same (latent) true reliability.
4. A specific advantage of DCERs seems to come from small sample size, short tests, and test with extreme difficulty levels and binary items. In these settings, the traditional conservative estimators (alpha, theta, and omega) may radically underestimate population reliability.
5. With binary items, all DCERs in the comparison seem to give almost an identical outcome that is notably higher than that given by the traditional estimators. The differences between DCERs are clearer with polytomous items.
6. Of the individual DCERs, those using *G* and *D* as the linking factor tend to be conservative with polytomous items, specifically if alpha and theta are used as the base. This is caused by the known characteristic of *G* and *D* to underestimate the item–score association in an obvious manner when the number of categories in the scale in an item exceeds 3–4. In these cases, instead of *G* and *D*, DCERs using dimension-corrected *G* and *D* (*G*_2_ and *D*_2_) as the linking factor give estimates with a magnitude close to the estimates by other estimators. Estimators using *D*_2_ as the linking factor tend to give more conservative outcomes than *G*_2_.
7. DCERs using *E*_*AC*_ as the linking factor offer a puzzle: although the magnitudes of the sample estimates are not higher than those given by the other DCERs, they tend to overestimate the *population* estimates using *E*_*AC*_ as the linking factor. This is specifically true when rho is used as the base with polytomous items. This uniquely reflects the relationship between the sample and population *E*_*AC*_. A large population rarely leads to deterministic or near-deterministic patterns between two variables; small samples are more prone to these patterns, and the magnitude of the estimates by *E*_*AC*_ in a sample tends to be higher than in the population.

The characteristics of different combinations of the base and the linking factor are discussed in the section that follows.

### Typology of Selected Deflation-Corrected Estimators of Reliability

Tables 2a,b summarize the typological characteristics of different combinations of the bases (alpha, theta, omega, and rho) and the weight factors (*R*_*PC*_, *R*_*REG*_, *G, D*, *G*_2_, *D*_2_, *R*_*AC*_, and *E*_*AC*_). Notably, all score variables discussed in the article (θ_*X*_, θ_*PC*_, θ_*FA*_, θ_*IRT*_, or θ_*NL*_) are not covered in this study; the raw score (θ_*X*_) was used in the simulation (of a comparison of other score variables; refer to Metsämuuronen, 2022a). The characteristics of the weight factors are studied elsewhere (e.g., Metsämuuronen, 2020a,b, 2021a,b, 2022b,d).

**Table 2a:** Typology of selected deflation-corrected estimators of reliability and their characteristics.

			**RPC**	**RREG**	**G & D**	**G2 & D2**
		General characteristics	• Reflects latent reliability, not strictly related to the observed score nor observed items • Leads to theoretical interpretation of reliability • Based on covariance • Suitable for binary and polytomous items • Not simple to calculate	• Reflects reliability of the observed score but uses non-observed items • Leads to partly theoretical interpretation of reliability • Based on regression model • Suitable for binary and polytomous items • Not simple to calculate	• Reflects reliability of observed score • Leads to practical interpretation of reliability • Based on probability • D more conservative than G • Suitable for binary items and polytomous items with < 3 categories • Simple to calculate manually	• Reflects reliability of the observed score but uses non-observed items • Leads to practical interpretation of reliability • Based on probability • Liberal nature; D_2_ more conservative than G_2_ • Suitable for binary and polytomous items • Simple to calculate manually
Base	Alpha	• Always underestimates population reliability • Very conservative in nature • Gives estimates even with small sample sizes • Reaches the perfect reliability (rel = 1) when wi = 1, and *σ_*i*_* = *σ_*j*_*	$\frac{k}{k-1}\left(1-\frac{\overset{k}{\underset{i\;=\;1}{\sum }}\sigma_{i}^{2}}{{\left(\overset{k}{\underset{i\;=\;1}{\sum }}\sigma_{i}RPC_{i\;\theta \;}\right)}^{2}}\right)$	$\frac{k}{k-1}\left(1-\frac{\overset{k}{\underset{i\;=\;1}{\sum }}\sigma_{i}^{2}}{{\left(\overset{k}{\underset{i\;=\;1}{\sum }}\sigma_{i}RREG_{i\;\theta \;}\right)}^{2}}\right)$	$\frac{k}{k-1}\left(1-\frac{\overset{k}{\underset{i\;=\;1}{\sum }}\sigma_{i}^{2}}{{\left(\overset{k}{\underset{i\;=\;1}{\sum }}\sigma_{i}G_{i\;\theta \;}\right)}^{2}}\right)$	$\frac{k}{k-1}\left(1-\frac{\overset{k}{\underset{i\;=\;1}{\sum }}\sigma_{i}^{2}}{{\left(\overset{k}{\underset{i\;=\;1}{\sum }}\sigma_{i}{G_{2}}_{i\;\theta \;}\right)}^{2}}\right)$
	Theta	• Maximizes alpha • Conservative nature • Gives estimates even with small sample sizes • Reaches the perfect reliability (rel = 1) when wi = 1	$\frac{k}{k-1}\left(1-\frac{1}{\overset{k}{\underset{i\;=\;1}{\sum }}RPC_{i\;\theta \;}^{2}}\right)$	$\frac{k}{k-1}\left(1-\frac{1}{\overset{k}{\underset{i\;=\;1}{\sum }}RREG_{i\;\theta \;}^{2}}\right)$	$\frac{k}{k-1}\left(1-\frac{1}{\overset{k}{\underset{i\;=\;1}{\sum }}G_{i\;\theta \;}^{2}}\right)$	$\frac{k}{k-1}\left(1-\frac{1}{\overset{k}{\underset{i\;=\;1}{\sum }}G_{2i\;\theta \;}^{2}}\right)$
	Omega	• Always higher than alpha • Least conservative nature • Gives estimates even with small sample sizes • Reaches the perfect reliability (rel = 1) when wi = 1	$\frac{{\left(\overset{k}{\underset{i\;=\;1}{\sum }}RPC_{i\;\theta \;}\right)}^{2}}{{\left(\overset{k}{\underset{i\;=\;1}{\sum }}RPC_{i\;\theta \;}\right)}^{2}+\overset{k}{\underset{g=1}{\sum }}\left(1-RPC_{i\;\theta \;}^{2}\right)}$	$\frac{{\left(\overset{k}{\underset{i\;=\;1}{\sum }}RREG_{i\;\theta \;}\right)}^{2}}{{\left(\overset{k}{\underset{i\;=\;1}{\sum }}RREG_{i\;\theta \;}\right)}^{2}+\overset{k}{\underset{g=1}{\sum }}\left(1-RREG_{i\;\theta \;}^{2}\right)}$	$\frac{{\left(\overset{k}{\underset{i\;=\;1}{\sum }}G_{i\;\theta \;}\right)}^{2}}{{\left(\overset{k}{\underset{i\;=\;1}{\sum }}G_{i\;\theta \;}\right)}^{2}+\overset{k}{\underset{g=1}{\sum }}\left(1-G_{i\;\theta \;}^{2}\right)}$	$\frac{{\left(\overset{k}{\underset{i\;=\;1}{\sum }}{G_{2}}_{i\;\theta \;}\right)}^{2}}{{\left(\overset{k}{\underset{i\;=\;1}{\sum }}{G_{2}}_{i\;\theta \;}\right)}^{2}+\overset{k}{\underset{g=1}{\sum }}\left(1-G_{2i\;\theta \;}^{2}\right)}$
	rho (maximal reliability)	• Maximizes omega • Liberal nature; may overestimate reliability with small sample sizes • Cannot be calculated if deterministic patterns even in one item • Cannot reach the perfect reliability (rel = 1) • Not the best option for small samples	$\frac{1}{1+\frac{1}{\overset{k}{\underset{i\;=\;1}{\sum }}\left(RPC_{i\;\theta \;}^{2}/\left(1-RPC_{i\;\theta \;}^{2}\right)\right)}}$	$\frac{1}{1+\frac{1}{\overset{k}{\underset{i\;=\;1}{\sum }}\left(RREG_{i\;\theta \;}^{2}/\left(1-RREG_{i\;\theta \;}^{2}\right)\right)}}$	$\frac{1}{1+\frac{1}{\overset{k}{\underset{i\;=\;1}{\sum }}\left(G_{i\;\theta \;}^{2}/\left(1-G_{i\;\theta \;}^{2}\right)\right)}}$	$\frac{1}{1+\frac{1}{\overset{k}{\underset{i\;=\;1}{\sum }}\left(G_{2i\;\theta \;}^{2}/\left(1-G_{2i\;\theta \;}^{2}\right)\right)}}$

*MEC-corrected estimators*.

**Table 2b:** Typology of selected deflation-corrected estimators of reliability and their characteristics; attenuation-corrected estimators.

			**Attenuation-corrected estimators;Weight wi**	
			**RAC**	**EAC**
		General characteristics	• Reflects reliability of the observed score but uses non-observed items • Leads to practical interpretation of reliability • Based on probability • May have a liberal nature • Tendency for slight overestimation with polytomous items • Safe to use with items with < 4 categories • Simple to calculate manually	• Reflects reliability of the observed score but uses non-observed items • Leads to practical interpretation of reliability • Based on probability • Very liberal nature • Tendency for overestimation with polytomous items • Safe to use with binary items • Simple to calculate manually
Base	Alpha	• Always underestimates population reliability • Very conservative in nature • Gives estimates even with small sample sizes • Reaches the perfect reliability (REL = 1) when wi = 1, and *σ_*i*_* = *σ_*j*_*	$\frac{k}{k-1}\left(1-\frac{\overset{k}{\underset{i\;=\;1}{\sum }}\sigma_{i}^{2}}{{\left(\overset{k}{\underset{i\;=\;1}{\sum }}\sigma_{i}RAC_{i\;\theta \;}\right)}^{2}}\right)$	$\frac{k}{k-1}\left(1-\frac{\overset{k}{\underset{i\;=\;1}{\sum }}\sigma_{i}^{2}}{{\left(\overset{k}{\underset{i\;=\;1}{\sum }}\sigma_{i}EAC_{i\;\theta \;}\right)}^{2}}\right)$
	Theta	• Maximizes alpha • Conservative nature • Gives estimates even with small sample sizes • Reaches the perfect reliability (REL = 1) when wi = 1	$\frac{k}{k-1}\left(1-\frac{1}{\overset{k}{\underset{i\;=\;1}{\sum }}RAC_{i\;\theta \;}^{2}}\right)$	$\frac{k}{k-1}\left(1-\frac{1}{\overset{k}{\underset{i\;=\;1}{\sum }}EAC_{i\;\theta \;}^{2}}\right)$
	Omega	• Always higher than alpha • Least conservative nature • Gives estimates even with small sample sizes • Reaches the perfect reliability (REL = 1) when wi = 1	$\frac{{\left(\overset{k}{\underset{i\;=\;1}{\sum }}RAC_{i\;\theta \;}\right)}^{2}}{{\left(\overset{k}{\underset{i\;=\;1}{\sum }}RAC_{i\;\theta \;}\right)}^{2}+\overset{k}{\underset{g=1}{\sum }}\left(1-RAC_{i\;\theta \;}^{2}\right)}$	$\frac{{\left(\overset{k}{\underset{i\;=\;1}{\sum }}EAC_{i\;\theta \;}\right)}^{2}}{{\left(\overset{k}{\underset{i\;=\;1}{\sum }}EAC_{i\;\theta \;}\right)}^{2}+\overset{k}{\underset{g=1}{\sum }}\left(1-EAC_{i\;\theta \;}^{2}\right)}$
	Rho (maximal reliability)	• Maximizes omega • Liberal nature; may overestimate reliability with small sample sizes • Cannot be calculated if deterministic patterns even in one item • Cannot reach the perfect reliability (rel < 1) • Not the best option for small samples	$\frac{1}{1+\frac{1}{\overset{k}{\underset{i\;=\;1}{\sum }}\left(RAC_{i\;\theta \;}^{2}/\left(1-RAC_{i\;\theta \;}^{2}\right)\right)}}$	$\frac{1}{1+\frac{1}{\overset{k}{\underset{i\;=\;1}{\sum }}\left(EAC_{i\;\theta \;}^{2}/\left(1-EAC_{i\;\theta \;}^{2}\right)\right)}}$

When it comes to the base of DCERs, the estimators based on alpha, theta, and omega are conservative; they tend to produce estimates that are underestimates of population reliability with small sample sizes. Estimators based on rho tend to be liberal; they tend to produce estimates that are overestimates of population reliability with small sample sizes. Estimators based on theta seem surprisingly stable, more stable than those by alpha and omega. Estimators based on rho are specifically vulnerable to deterministic patterns. In these patterns, estimates by rho cannot be calculated because of the undefined division by zero. Also, the estimates by rho are unstable with a near-deterministic pattern even in one item. These patterns are expected with small sample sizes. Hence, DCERs based on rho may not be suggested to be used with small sample sizes.

When it comes to weighting factors, *R*_*PC*_ and *R*_*REG*_ reflect a correlation between unobservable, theoretical constructions. Hence, DECRs using these coefficients as linking factors may lead to a kind of *theoretical* reliability that is not related to the factual score variable (refer to the critique by Chalmers, 2017). From this viewpoint, estimators based on *G* and *D* lead to more practical interpretations of reliability. That is, because *G* and, specifically, *D* strictly indicate the proportion of logically ordered test-takers in a test item after they are ordered by the score (refer to Metsämuuronen, 2021b), the DCERs using *G* or *D* reflect the proportion of logically ordered test-takers in all test items as a whole. For example, if the average *D* of all item–score correlations in a specific dataset is 0.7, it means that 85% of the test takers, that is, *p* = 0.5 × 0.70 + 0.5 = 0.85 (refer to Metsämuuronen, 2021b), are logically ordered in all items as a whole after they are ordered by the score. Because of their conservative nature with polytomous items having more than three categories, DCERs based on *G* and *D* are suggested for tests with binary items and with polytomous items having less than four categories. The dimension-corrected versions of *G* and *D* (*G*_2_ and *D*_2_) can be used for binary and polytomous items and in a binary case, *G* = *G*2 and *D* = *D*_2_.

Of the DCERs using attenuation-corrected estimators of correlation (*R*_*AC*_ and *E*_*AC*_) as the linking factor, those using *R*_*AC*_ are more conservative than those using *E*_*AC*_. This follows strictly from the behavior of *R*_*AC*_ and *E*_*AC*_: except for the binary case where *R*_*AC*_ and *E*_*AC*_ give identical estimates, the estimates by *E*_*AC*_ tend to be higher than those by *R*_*AC*_ (refer to, e.g., Metsämuuronen, 2022d). Both seem to be somewhat liberal with small sample sizes especially with polytomous items, although the factual estimates do not seem to differ notably from the estimates by other DCERs. With binary items, ACERs tend to produce largely the same estimates as MCERs.

Based on the simulation, some initial recommendations concerning the usability of the DCERs may be summarized as follows; obviously, more specified simulations are needed, and these are discussed in the next section.

1. With small sample sizes (*n* < 200), using estimators based on rho is not recommendable; all DCERs based on rho as well as the traditional estimators tend to give overestimates with small sample sizes.
2. With binary items, all DCERs based on the conservative estimators (alpha, theta, and omega) give more plausible estimates than the traditional estimators; the difference is in the interpretation of the linking factor. Using *R*_*PC*_ or *R*_*REG*_ leads to “theoretical reliability” as a benchmark for the traditional one and using *G* or *D* (and *G*_2_ or *D*_2_) leads to practical interpretation of the logical order of the test-takers; all these refer to the discrimination power of the score. Using *R*_*AC*_ or *E*_*AC*_ may give an interpretation closer to the original *Rit*, that is, attenuation-corrected alpha, theta, omega, or rho. Notably, with binary items, *R*_*AC*_ and *E*_*AC*_ produce identical outcomes.
3. With polytomous items, DCERs using *G* and *D* are not recommended to be used is the number of categories exceeds 3 (*D*) or 4 (*G*), or, if used, the estimates may be very conservative—the magnitude of the estimates may be even more deflated than of those by the traditional alpha. Specifically, if the number of categories in the score is small but the sample size is large, *D* tends to be affected by the large number of tied cases and tends to underestimate the correlation, which is also reflected in the estimates of reliability. With polytomous items, using *G*_2_ or *D*_2_ seems to give estimates whose magnitude is closer to those by *R*_*PC*_ or *R*_*REG*_. However, using *G*_2_ and *EAC* may give a liberal estimate in comparison with *R*_*PC*_, *R*_*REG*_, *D*_2_, and *R*_*AC*_.
4. If alpha and theta are used, where the traditional item–score correlation is originally used as default, as the bases for DCERs, attenuation-corrected *Rit* (*R*_*AC*_) could be a natural alternative for *Rit*. Then, the “attenuation corrected alpha” or “attenuation corrected theta” could be reported as a benchmark as a side of the traditional alpha or theta. Using *E*_*AC*_ could enhance the outcome by also allowing non-linearity in the association between items and score. Obviously, the other alternative estimators could also be used; then, we could report “MEC-corrected alpha” or “deflation-corrected alpha” as a benchmark.
5. If using omega and rho as the bases for DCERs, three options may be worth considering. First, a renewed process of producing factor loadings may be considered; for DCERs, the factor loadings should be some of the alternative estimators of item–score correlation instead of (essentially) *Rit*. Second, another option to estimate the reliability of the factor score variables would be to estimate just the factor score variable by traditional factor analysis to produce an “optimal linear combination” and to use alternative estimators of item–score correlation in the DCERs irrespective of factor loadings. Third, in line with the general approach used in the article, the formulae of omega and rho could be used in DCERs to estimate the reliability of various types of score variables irrespective of the factor analysis. Systematic studies on these options would be beneficial.

### Practical Calculation of DCERs

To give a practical example of calculating the DCERs discussed in this article, a specific national-level dataset with exceptionally easy items (n = 7,770) discussed by Metsämuuronen (2022b; 2022f; 2022g; originally in Metsämuuronen and Ukkola, 2019) and referred to in sections “From prediction formulae to coefficient alpha” and “From alpha, theta, omega, and rho to deflation-corrected reliability” is used here as an example. Originally, the test was a screening test of proficiency in the language used in the factual test; only test-takers with second language status were expected to make mistakes in the test items. Descriptive statistics of the dataset are collected in Table 3a, principal component and factor loadings for the traditional theta, omega, and rho in Table 3b, estimates of item–score correlation by selected estimators of correlation in Table 3c, and derivatives of the correlations for the traditional and deflation-corrected coefficients of alpha in Table 3d. Estimates of reliability are collected in Table 3e.

**Table 3a:** Descriptive statistics of the test items from Metsämuuronen and Ukkola (2019) (*N* = 7,770).

**Item (g)**	**Range**	**Mean**	***p***	**Std. deviation**	**Variance**
g1	0–1	0.96	0.96	0.186	0.0348
g2	0–1	0.98	0.98	0.126	0.0160
g3	0–1	0.99	0.99	0.088	0.0078
g4	0–1	0.91	0.91	0.287	0.0824
g5	0–2	1.78	0.89	0.610	0.3715
g6	0–1	0.98	0.98	0.122	0.0150
g7	0–2	1.97	0.985	0.211	0.0446
g8	0–2	1.98	0.99	0.169	0.0285
SUM					0.6004
Score	3–11	10.57	0.961	0.875	0.7650

**Table 3b:** Principal component and factor loadings.

	**Principal component loadings and derivatives**		**Factorloadings and derivatives**			
**Item**	**λ_PC_**	** $\lambda_{\text{PC}}^{2}$ **	**λ_MLE_**	** $\lambda_{\text{MLE}}^{2}$ **	**1–$\lambda_{\text{MLE}}^{2}$**	**$\lambda_{\text{MLE}}^{2}$/(1–$\lambda_{\text{MLE}}^{2}$)**
g1	0.447	0.200	0.276	0.076	0.924	0.082
g2	0.430	0.185	0.260	0.068	0.932	0.073
g3	0.605	0.366	0.471	0.222	0.778	0.285
g4	0.468	0.219	0.291	0.085	0.915	0.093
g5	0.204	0.042	0.111	0.012	0.988	0.012
g6	0.375	0.141	0.213	0.045	0.955	0.048
g7	0.288	0.083	0.160	0.026	0.974	0.026
g8	0.633	0.401	0.512	0.262	0.738	0.355
SUM		1.636	2.294		7.204	0.974

**Table 3c:** Estimators of correlation between the item and raw score.

**item**	**Rit**	**RPC**	**RREG**	**D**	**G**	**D2**	**G2**	**RAC**	**EAC**
g1	0.351	0.677	0.436	0.791	0.857	0.791	0.857	0.551	0.551
g2	0.268	0.618	0.375	0.779	0.846	0.779	0.846	0.489	0.489
g3	0.283	0.696	0.408	0.858	0.911	0.858	0.911	0.603	0.603
g4	0.458	0.736	0.529	0.789	0.834	0.789	0.834	0.603	0.603
g5	0.746	0.931	0.732	0.952	0.979	0.958	0.982	0.921	0.923
g6	0.260	0.602	0.364	0.766	0.831	0.766	0.831	0.477	0.477
g7	0.327	0.702	0.425	0.832	0.897	0.943	0.976	0.568	0.567
g8	0.373	0.760	0.457	0.877	0.924	0.961	0.983	0.680	0.693

**Table 3d:** Derivatives of the estimators of correlation between an item and a raw score.

**Item**	**VAR(g)**	**Rit × s**	**RPC × s**	**D × s**	**G × s**	**D2 × s**	**G2 × s**	**RAC × s**	**EAC × s**
g1	0.035	0.065	0.126	0.147	0.160	0.147	0.160	0.103	0.103
g2	0.016	0.034	0.078	0.098	0.107	0.098	0.107	0.062	0.062
g3	0.008	0.025	0.061	0.076	0.080	0.076	0.080	0.053	0.053
g4	0.082	0.131	0.211	0.226	0.239	0.226	0.239	0.173	0.173
g5	0.372	0.455	0.568	0.580	0.597	0.584	0.598	0.561	0.562
g6	0.015	0.032	0.074	0.094	0.102	0.094	0.102	0.058	0.058
g7	0.045	0.069	0.148	0.176	0.189	0.199	0.206	0.120	0.120
g8	0.028	0.063	0.128	0.148	0.156	0.162	0.166	0.115	0.117
SUM	0.600	0.874	1.395	1.546	1.630	1.587	1.658	1.245	1.248

**Table 3e:** Estimates of reliability.

	**Traditionalestimator**	**DCERs with alternative weight factors and raw score (θ_X_)**							
**Base**	**Traditionalweight (score)**	**RPC**	**RREG**	**D**	**G**	**D2**	**G2**	**RAC**	**EAC**
Alfa	0.2450 (θ_X_)	0.7901	0.4196	0.8556	0.8846	0.8703	0.8934	0.7004	0.7025
Theta	0.4444 (θ_PC_)	0.8686	0.5200	0.9368	0.9610	0.9494	0.9684	0.7779	0.7802
Omega	0.4221 (θ_MLE_)	0.8952	0.6925	0.9473	0.9669	0.9572	0.9729	0.8310	0.8323
Rho	0.4934 (θ_MLE_)	0.9287	0.7353	0.9605	0.9795	0.9757	0.9891	0.9012	0.9031

For the traditional alpha, theta, omega, and rho, their original score variable is used: a raw score for alpha, a principal component (PC) score for theta, and an ML estimate (MLE) of the factor score for omega and rho. For DCERs, the raw score is used as the manifestation of the latent variable; Metsämuuronen (2022f) shows examples of using PC and factor scores in calculations.

Using Tables 3a,d and eq. (2), the estimate of reliability by the traditional alpha is ${\hat{\rho }}_{\alpha }=\frac{k}{k-1}\left(1-\frac{\overset{k}{\underset{i\;=\;1}{\sum }}\sigma_{i}^{2}}{{\left(\overset{k}{\underset{i\;=\;1}{\sum }}\sigma_{i}\rho_{i\;\theta \;X}\right)}^{2}}\right)=\frac{8}{7}\left(1-\frac{0.6004}{0.874^{2}}\right)=0.245$. Correspondingly, using Table 3b and eqs. (6), (8) and (9), the estimate by theta is ${\hat{\rho }}_{TH}=\frac{k}{k-1}\left(1-\frac{1}{\overset{k}{\underset{i\;=\;1}{\sum }}\lambda_{i\;\theta \text{PC}}^{2}}\right)=\frac{8}{7}\left(1-\frac{1}{1.636}\right)=0.444$, the estimate by omega is ${\hat{\rho }}_{\omega }=\frac{{\left(\overset{k}{\underset{i\;=\;1}{\sum }}\lambda_{i\;\theta \text{MLE}}\right)}^{2}}{{\left(\overset{k}{\underset{i\;=\;1}{\sum }}\lambda_{i\;\theta \text{MLE}}\right)}^{2}+\overset{k}{\underset{i\;=\;1}{\sum }}\left(1-\lambda_{i\;\theta \text{MLE}}^{2}\right)}=\frac{2.294^{2}}{2.294^{2}+7.204}=0.422$, and the estimate by rho is ${\hat{\rho }}_{MAX}=\frac{1}{1+\frac{1}{\overset{k}{\underset{i\;=\;1}{\sum }}\left(\lambda_{i\;\theta \text{MLE}}^{2}/\left(1-\lambda_{i\;\theta \text{MLE}}^{2}\right)\right)}}=\frac{1}{1+\frac{1}{0.974}}=0.493$.

Similarly, the estimates by DCERs can be calculated using eqs. (22) to (25) by applying different weight factors.^4^ If RPC is used as the weight factor, deflation-corrected alpha, as an example, gives an estimate of ${\hat{\rho }}_{\alpha \text{\_}RPCi\;\theta \text{X}}=\frac{k}{k-1}\left(1-\frac{\overset{k}{\underset{i\;=\;1}{\sum }}\sigma_{i}^{2}}{{\left(\overset{k}{\underset{i\;=\;1}{\sum }}\sigma_{i}RPC_{i\;\theta \text{X}}\right)}^{2}}\right)=\frac{8}{7}\left(1-\frac{0.6004}{1.395^{2}}\right)=0.790$ and, if G is used as the linking factor, ${\hat{\rho }}_{\alpha \text{\_}Gi\;\theta \text{X}}=\frac{k}{k-1}\left(1-\frac{\overset{k}{\underset{i\;=\;1}{\sum }}\sigma_{i}^{2}}{{\left(\overset{k}{\underset{i\;=\;1}{\sum }}\sigma_{i}G_{i\;\theta \text{X}}\right)}^{2}}\right)=\frac{8}{7}\left(1-\frac{0.6004}{1.630^{2}}\right)=0.885$. In both cases, the message is the same: the estimate by the traditional alpha is radically deflated; instead of 0.24, the level of reliability is most probably closer to 0.79– 0.85. Deflation-corrected thetas vary, 0.778–0.968, deflation-corrected omegas vary, 0.831–0.973, and deflation-corrected rhos vary, 0.901–0.989. These are notably higher than the deflated traditional theta (0.444), omega (0.422), and rho (0.493). In these kinds of datasets with extreme difficulty levels, DCERs may give a notable advantage in estimating the true reliability.

### Known Limitations and Suggestions for Further Studies

The paradigm of deflation-correction in the estimates of reliability is still in the early stage. We do not know yet much about the new types of estimators of reliability. The simulation conducted in this article has obvious limits: only small sample sizes were used, the latent reliability was not controlled as is a norm in Monte Carlo simulations, the score variables was restricted only to raw score, tests with more than 30 and less than 10 categories in the score were missing, and no tests with extreme difficulty level or very short tests were not included in the simulation. Further investigation of such settings would be beneficial. Also, by far, only limited estimators of correlations as alternatives for *Rit* have been studied.

One obvious need of the new paradigm is to create a sound theoretical base for DCERs. From this viewpoint, DCERs based on omega and rho may be easier to argue for: the theoretical base discussed in eqs. (16) to (21) may be used as a sufficient conceptual or theoretical basis for DCERs. However, many traditional estimators are strictly based on variances, observed variance and error variance, leading to use of the traditional item–score correlation, which leads to deflation. The alternative estimators discussed in this article are mainly short-cuts replacing *Rit* in the process. However, if we want to create or develop an estimator such as ρ_BS_, ρ_FR_, ρ_KR20_, and ρ_α_ from scratch and to avoid embedding *Rit* in the formulae, would the estimator still look like in the traditional formulae?

Another obvious restriction of the study is that only estimators from the classical test theory were discussed. A relevant question is, how applicable the results would be with estimators of reliability within Generalizability Theory (G-Theory; chronologically, e.g., Cronbach et al., 1972; Shavelson et al., 1989; Shavelson and Webb, 1991; Brennan, 2001, 2010; Vispoel et al., 2018a,b; Clayson et al., 2021), confirmatory factor analysis (CFA) or structural equation modeling (SEM refer to, e.g., Raykov and Marcoulides, 2006; Green and Yang, 2009b), and IRT and Rasch modeling (refer to estimators in e.g., Verhelst et al., 1995; Holland and Hoskens, 2003; Kim and Feldt, 2010; Cheng et al., 2012; Kim, 2012; Milanzi et al., 2015)? Except for the estimators developed for CFA and SEM analysis, in all cases, the possible deflation in the estimates is not as obvious as with the classical estimators, because the latter can be expressed using Rit and principal and factor loadings that are obviously deflated. Estimators using factor loading (as is a tradition in the basic CFA and SEM) are most probably prone to severe deflation because factor loadings are prone to deflation.

In G-Theory, the challenge is that, first, *two* types of estimators are used: the generalizability coefficient and the dependability coefficient; the former is low when interindividual rankings are inconsistent, and the latter is low when measurements from same individuals are inconsistent (refer to condensed discussion in Clayson et al., 2021). Although the former is more comparable with classical estimators such as coefficient alpha, we do not know the possible *mechanics* of deflation in these estimators. Second, in estimating the reliability within the framework of G-Theory, variance components are radically more complicated than when using classical estimators (refer to Brennan, 2001; Vispoel et al., 2018a; Clayson et al., 2021). Furthermore, Vispoel et al. (2018a) noted that failing to consider each source of measurement variance can result in overestimation of reliability. Hence, systematic theoretical and empirical studies are needed to confirm the possible sources of deflation in estimates by G-Theory.

In Rasch and IRT modeling, the estimation of reliability is often based on such concepts as “person separation” in Rasch models (Andrich and Douglas, 1977; Andrich, 1982; Wright and Masters, 1982) or “information function” in wider IRT models (refer to, e.g., McDonald, 1999; Cheng et al., 2012; Milanzi et al., 2015). These are not necessarily prone to deflation in an obvious manner. However, what *is* known is that the estimator called Accuracy of Measurement (MAcc) discussed by Verhelst et al. (1995) with a one-parameter logistic model tends to be severely affected by the form of distribution of the score; when the score variable is notably skewed, that is, when the test is either extremely easy or difficult to the target population, the estimates may even be far off the range of reliability (refer to the empirical examples in Metsämuuronen, 2022g).^5^ If we assume that the estimates may be deflated in the estimators of reliability within the IRT modeling, two possible sources would be worth studying: the formulae themselves may not be effective or the estimates for item discrimination (*a*-parameter) often needed in the estimation would be deflated. With MAcc, it seems obvious that the operationalization of error variance of the score should be reconsidered (refer to Metsämuuronen, 2022g). Systematic studies, in this regard, would be beneficial.

Using score variance as a basis of reliability within the classical test theory leads easily to item–score correlation, which leads to deflation. If we want to avoid using variances as the base for reliability, one option for reconceptualizing reliability discussed by Metsämuuronen (2022a) is to define “perfect reliability” (*REL* = 1) as a condition where the score can discriminate test-takers in all items in a deterministic manner in the spirit of Guttman's scalogram (Guttman, 1950). This is related to the estimators of reliability within the non-parametric IRT modeling (NIRT; Mokken, 1971) where the coefficient H by Loevinger (1948) indicates homogeneity in the dataset and deviance from the deterministic pattern or so-called “Guttman-homogeneity” (refer to Molenaar and Sijtsma, 1984). This could lead to (correctly) detecting perfect reliability by DCERs based on theta and omega using *R*_*PC*_, *G*, *G*_2_, *R*_*AC*_, and *E*_*AC*_ as the linking factors (see eqs. 22–25). *D* could be used as the linking factor in defining restrictions in Monte Carlo simulations: 90% of logically ordered test-takers in all items, after they are ordered by the score, lead to omegaD = 0.9^2^ = 0.81 and 80% to omegaD = 0.8^2^ = 0.64. Other options could be based on “sufficiency of information” (Smith, 2005), “person separation” (Andrich and Douglas, 1977; Andrich, 1982; Wright and Masters, 1982; refer also to “Rasch reliability” in Linacre, 1997; Clauser and Linacre, 1999), the “information function” (refer to, e.g., McDonald, 1999; Cheng et al., 2012; Milanzi et al., 2015) discussed in item response theory (IRT) settings, or “person-fit” within the paradigm of NIRT (refer to, e.g., Meijer et al. (1995).

The final note for further studies comes from the fact that the extended family of DCERs also includes estimators such as the ordinal alpha and ordinal theta proposed by Zumbo et al. (2007). Other less known estimators may also be included. Ordinal alpha and theta are based on changing the inter-item matrices of PMCs by matrices of *R*_*PC*_*s* instead of changing the linking factor itself. It is expected that the estimates by ordinal alpha and theta would be identical with those by the theta RPC and alpha RPC discussed in this article, because the estimates using the traditional formula of alpha and an alternative computational form using the matrices of inter-item correlations are identical. However, it is not known whether estimates by factor analysis using the matrix of RPCs would lead to factor loadings that are *R*_*PC*_s. If the estimates are identical, it would be easy to obtain DCERs based on omega and rho using traditional procedures simply by changing the inter-item matrix of *Rit*s to the matrix of *R*_*PC*_s, *G*s, or *D*s, for instance. However, if the loadings are still (essentially) *Rit*s, calculated using the mechanics of PMC, it could be valuable to develop new procedures for FA/PCA so that the factor loadings needed in DCERs would be, factually, *R*_*PC*_s, *G*s, or *D*s, for instance, as discussed above.

## Data Availability Statement

Publicly available datasets were analyzed in this study. This data can be found here: http://dx.doi.org/10.13140/RG.2.2.10530.76482 http://dx.doi.org/10.13140/RG.2.2.17594.72641. http://dx.doi.org/10.13140/RG.2.2.30493.03040 http://dx.doi.org/10.13140/RG.2.2.27971.94241.

## Author Contributions

The author confirms being the sole contributor of this work and has approved it for publication.

## Conflict of Interest

The author declares that the research was conducted in the absence of any commercial or financial relationships that could be construed as a potential conflict of interest.

## Publisher's Note

All claims expressed in this article are solely those of the authors and do not necessarily represent those of their affiliated organizations, or those of the publisher, the editors and the reviewers. Any product that may be evaluated in this article, or claim that may be made by its manufacturer, is not guaranteed or endorsed by the publisher.

## Footnotes

^1^An anonymous reviewer raised the challenge of simplified dimensionality (as part of the error in measurement modeling) as possibly having a profound effect on the underestimation of reliability; if the multidimensionality in the measurement instrument would be considered, the reliability would be profoundly higher (refer to, e.g., McNeish, 2017). From the deflation viewpoint, however, the effect of dimensionality may be less profound, although more studies would enrich the discussion. Namely, even if the multidimensionality would be considered but the items are of extreme difficulty levels in a dimension (as is usual in the achievement testing), the fact remains that the deflation in factor loadings and item score correlations is way more radical than the advance we get from dimensionality. The deflation in factor loadings and item score correlations is discussed in section “PMC as the root cause for the deflation in reliability.

^2^Notably, *RPC* cannot reach a perfect 1. With enhanced procedures of the estimation by adding a very small number like 10^−50^ to each element of the logarithm and when the embedded PMC ≈ 1 such as 0.99999999, *RPC* ≈ 1.

^3^The dataset of individual items (*n* = 14,880) including several indicators of item–score association is available in CSV format at http://dx.doi.org/10.13140/RG.2.2.10530.76482 and in SPSS format at http://dx.doi.org/10.13140/RG.2.2.17594.72641. The dataset of reliabilities (*n* = 1,440) is available in CSV format at http://dx.doi.org/10.13140/RG.2.2.30493.03040 and in SPSS format at http://dx.doi.org/10.13140/RG.2.2.27971.94241.

^4^The derivatives of the coefficients of correlation for DCERs based on theta, omega, and rho are not seen in Tables 3b–d. These are, however, easy to calculate from the original correlations in Table 3c, in the same manner done in Table 3b. Estimates by RREG seem notably lower than the other estimates of correlation; in what follows, these are taken as underestimates.

^5^In the specific dataset of achievement in the instruction language of a test in mathematics (*n* = 7,770) with extremely easy items and radically non-normal distribution discussed by Metsämuuronen (2022a,c,f) and re-analyzed above, the estimate by MAcc (Verhelst et al., 1995, pp. 99–100) was obviously out of range (MAcc = −5.89), while the traditional alpha = 0.245, theta = 0.444, omega = 0.422, and rho = 0.493, although deflated, were within the range of reliability. In April 2022, this specific dataset was re-analyzed by the teams of Milanzi et al. (2015) and Cheng et al. (2012) using the estimators they suggested in their articles. The results will be reported later. In this case, it would also be informative to apply Foster's (2021) enhanced KR20 developed for non-normal datasets such as exponential distributions in the score.
