# Supplementary material for: Typology of Deflation-Corrected Estimators of Reliability
Source: Front Psychol. 2022 Jul 18;13:891959. doi: 10.3389/fpsyg.2022.891959 (PMC9341485; doi:10.3389/fpsyg.2022.891959)
Supplement: Supplementary file 1 [file Data_Sheet_1.docx]

# Typology of deflation-corrected estimators of reliability

# Appendix 1. Estimation of the coefficients of correlation used in DCERs

## Polychoric correlation (R_PC_)

In traditional software packages such as IBM SPSS (IBM, 2017), the syntax for *R_PC_* is not available although some macros are (see Lorenzo-Seva & Ferrando, 2015). In SAS, the command PROC CORR provides *R_PC_*. Correspondingly, in RStudio, as an example, *R_PC_* is calculated by [*CorPolychor*](https://rdrr.io/cran/DescTools/man/CorPolychor.html)*(x, y, ML = FALSE, control = list(), std.err = FALSE, maxcor=.9999)*## S3 method for class 'CorPolychor' *print(x, digits = max(3, getOption(*"digits"*) - 3), ...)* (see <https://rdrr.io/cran/DescTools/man/CorPolychor.html>). In the article, *R_PC_* was calculated by using the simplified procedure by Zaiontz (2022) and MS-Excel software.

## r-biserial and r-polyreg correlation (R_REG_)

For *R_REG_*, *β_i_* is the slope parameter of the probit regression model $P\left(x_{i}\leq1\left|y\right.\right)=\Phi\left(a_{i}-\beta_{i}y\right)$ where Φ is the standard normal cumulative distribution function and *a_i_* and *β_i_* are intercept and slope parameters (see Moses, 2017). The *β*-value can be calculated, for example, in IBM SPSS software using the syntax

*GENLIN g (ORDER=ASCENDING) WITH X /MODEL X*

*DISTRIBUTION=MULTINOMIAL*

*LINK=CUMPROBIT /CRITERIA METHOD=FISHER /PRINT SOLUTION.*

After the ML estimate of *β* is computed, *R_REG_* is calculated as $\rho_{REG}=\frac{\beta\sigma_{X}}{\sqrt{\beta^{2}\sigma_{X}^{2}+1}}$, where $\sigma_{X}^{2}$ is the estimated population variance of the score variable *X*. In the article, RREG was calculated manually after *β* and $\sigma_{X}^{2}$ were estimated by using SPSS.

## G and G_2_

In traditional software packages such as IBM SPSS, for instance, the syntax for *G* is CROSSTABS /TABLES=item BY Score /STATISTICS=GAMMA. In SAS, the command PROC FREQ provides *G* by specifying the TEST statement by GAMMA, SMDCR options. Correspondingly, in RStudio *G* is calculated by [*GoodmanKruskalGamma*](https://rdrr.io/cran/DescTools/man/GoodmanKruskalGamma.html)*(x, y = NULL, conf.level = NA, ...)* (see <https://rdrr.io/cran/DescTools/man/>). For the empirical section, the estimates by *G*_2_ are calculated manually based on the observed *G* and *df*(*g*) based on Metsämuuronen (2021).

The computational form of dimension-corrected *G* (*G*_2_) is$G_{2}=G\times\left(1+\left(1-abs\left(G\right)\right)\times A\right)$, where *G* is the observed value of *G* and $A=\left(\frac{df\left(g\right)-1}{df\left(g\right)}\right)^{3}$, where *df*(*g*) = (number of categories in the item – 1).

## D and D_2_

In traditional software packages such as IBM SPSS, for instance, the syntax for *D* is CROSSTABS /TABLES=item BY Score /STATISTICS=D. In SAS, the command PROC FREQ provides *D* by specifying the TEST statement by D, SMDCR options. Correspondingly, in RStudio, *D* is calculated by [*SomersDelta*](https://rdrr.io/cran/DescTools/man/SomersDelta.html)*(x, y = NULL, direction = c(*"row"*,* "column"*), conf.level = NA, ...)* (see <https://rdrr.io/cran/DescTools/man/>). For the empirical section, the estimates by *D*_2_ are calculated manually based on the observed *D* and *df*(*g*) based on Metsämuuronen (2021).

The computational form of (corrected) dimension-corrected *D* (*D*_2_) is

$D_{2}=D\times\left(1+\left(1-abs\left(D\right)\right)\times A\right)$, where *D* is the observed value of $D\left(g\left|X\right.\right)$ and $A=\left(\frac{df\left(g\right)-1}{df\left(g\right)}\right)^{3}$.

## Attenuation-corrected PMC and eta (RAC and EAC)

*RAC* is the proportion of the observed item–score correlation ($\rho_{gX}^{Obs}$) of maximal correlation ($\rho_{gX}^{Max}$) possible to obtain with the observed *g* and *X:* $\rho_{AC}=\frac{\rho_{gX}^{Obs}}{\rho_{gX}^{Max}}=\frac{\rho_{gX}}{\rho_{gX}^{Max}}$(Metsämuuronen, 2022a). *EAC* is the proportion of observed *eta* ($\eta_{g|X}^{Obs}$) and the maximal *eta* ($\eta_{g|X}^{Max}$) possible to obtain given the variance of the score: $\eta_{AC}=\frac{\eta_{g|X}^{Obs}}{\eta_{g|X}^{Max}}=\frac{\eta\left(g|X\right)}{\eta_{g|X}^{Max}}$(Metsämuuronen, 2022b).

The maximum values of both *Rit* and *eta* in the given dataset are obtained when the correlation is calculated between variables *g* and *X* after they are ordered *independently*. In the traditional software packages such as IBM SPSS, for instance, the syntax for *eta* is CROSSTABS /TABLES=item BY Score /STATISTICS=ETA. In SAS, the positive values of *eta* can be found by taking square of eta squared after PROC GLM with option EFFECTSIZE. Correspondingly, in RStudio, eta is calculated by [*eta*](https://rdrr.io/cran/ryouready/man/eta.html)*(x, y, breaks =* [*NULL*](https://rdrr.io/r/base/NULL.html)*, na.rm =* [*FALSE*](https://rdrr.io/r/base/logical.html)) (see <https://rdrr.io/cran/ryouready/man/eta.html>). For the maximal *Rit* and *eta*, in R, the variables (vectors) can be sorted by a command *sort (x) #.* For the empirical section, *RAC* and *EAC* were calculated manually by using a general spreadsheet software package. For correct values of *eta* also including the negative values, a simple transformation *sign*(*Rit*)×*eta* suggested by Metsämuuronen (2022) was used in the estimation.

## References

IBM. (2017). *IBM SPSS Statistics 25 Algorithms*. IBM. https://www.ibm.com/docs/en/SSLVMB_25.0.0/pdf/en/IBM_SPSS_Statistics_Algorithms.pdf (Accessed June 26, 2022).

Lorenzo-Seva, U., & Ferrando, P.J. (2015). POLYMAT-C: a comprehensive SPSS program for computing the polychoric correlation matrix. *Behavior Research Methods, 47***,** 884–889.. <https://doi.org/10.3758/s13428-014-0511-x>

Metsämuuronen J. (2021). Goodman–Kruskal gamma and dimension-corrected gamma in educational measurement settings. *International Journal of Educational Methodology, 7*(1), 95–118. <https://doi.org/10.12973/ijem.7.1.95>

Metsämuuronen, J. (2022a). Attenuation-corrected reliability and some other MEC-corrected estimators of reliability. *Applied Psychological Measurement* (in printing)

Metsämuuronen, J. (2022b). Artificial systematic attenuation in eta squared and some related consequences. Attenuation-corrected eta and eta squared, negative values of eta, and their relation to Pearson correlation. *Behaviormetrika,* <https://doi.org/10.1007/s41237-022-00162-2>

Moses, T. (2017). A review of developments and applications in item analysis. In R. Bennett & M. von Davier (Eds.), *Advancing human assessment*. *The methodological, psychological and policy contributions of ETS* (pp. 19–46). Educational Testing Service. Springer Open. <https://doi.org/10.1007/978-3-319-58689-2_2>

Zaionts, C. (2021) *Real Statics Using Excel.* Polychoric Correlation using Solver. <http://www.real-statistics.com/correlation/polychoric-correlation/polychoric-correlation-using-solver/> (accessed July 6, 2022)

# Appendix 2. Characteristics of DCERs

| weight | *Rit* | *λ_i_*_(PC)_ | *λ_i(ML)_* | *λ_i(ML)_* |  | RPC | RPC | RPC | RPC |  | RREG | RREG | RREG | RREG |
| --- | --- | --- | --- | --- | --- | --- | --- | --- | --- | --- | --- | --- | --- | --- |
| score | θ_X_ | θ_PC_ | θ_FA_ | θ_FA_ |  | θ_X_ | θ_X_ | θ_X_ | θ_X_ |  | θ_X_ | θ_X_ | θ_X_ | θ_X_ |
| base | Alpha | Theta | Omega | Rho |  | Alpha | Theta | Omega | Rho |  | Alpha | Theta | Omega | Rho |
| sample size | traditional estimates | | | |  | mean | | | |  | mean | | | |
| 25 (n = 360) | 0.815 | 0.835 | 0.819 | 0.871 |  | 0,867 | 0,878 | 0,911 | 0,931 |  | 0,875 | 0,886 | 0,916 | 0,931 |
| 50 (n = 360) | 0.859 | 0.866 | 0.862 | 0.881 |  | 0,898 | 0,902 | 0,929 | 0,937 |  | 0,889 | 0,894 | 0,923 | 0,931 |
| 100 (n = 360) | 0.863 | 0.867 | 0.865 | 0.877 |  | 0,900 | 0,902 | 0,930 | 0,936 |  | 0,888 | 0,890 | 0,921 | 0,927 |
| 200 (n = 360) | 0.864 | 0.866 | 0.865 | 0.872 |  | 0,901 | 0,903 | 0,931 | 0,934 |  | 0,889 | 0,890 | 0,922 | 0,925 |
| Total | 0.850 | 0.858 | 0.854 | 0.875 |  | 0,891 | 0,896 | 0,925 | 0,935 |  | 0,885 | 0,890 | 0,920 | 0,928 |
| N | 1440 | 1440 | 1394 | 1384 |  | 1440 | 1440 | 1440 | 1418 |  | 1440 | 1440 | 1440 | 1421 |
|  |  |  |  |  |  |  |  |  |  |  |  |  |  |  |
|  | Std. Deviation | | | |  | Std. Deviation | | | |  | Std. Deviation | | | |
| sample size | Alpha | Theta | Omega | Rho |  | Alpha | Theta | Omega | Rho |  | Alpha | Theta | Omega | Rho |
| 25 (n = 360) | 0.073 | 0.062 | 0.075 | 0.059 |  | 0,064 | 0,063 | 0,038 | 0,035 |  | 0,053 | 0,053 | 0,034 | 0,029 |
| 50 (n = 360) | 0.037 | 0.036 | 0.037 | 0.035 |  | 0,037 | 0,037 | 0,021 | 0,021 |  | 0,035 | 0,036 | 0,022 | 0,021 |
| 100 (n = 360) | 0.025 | 0.025 | 0.025 | 0.025 |  | 0,033 | 0,034 | 0,016 | 0,017 |  | 0,029 | 0,030 | 0,017 | 0,017 |
| 200 (n = 360) | 0.022 | 0.021 | 0.021 | 0.021 |  | 0,026 | 0,027 | 0,014 | 0,014 |  | 0,026 | 0,027 | 0,014 | 0,014 |
| Total  (n = 1,440) | 0.049 | 0.042 | 0.047 | 0.037 |  | 0,045 | 0,044 | 0,026 | 0,023 |  | 0,038 | 0,038 | 0,023 | 0,021 |
|  |  |  |  |  |  |  |  |  |  |  |  |  |  |  |
|  |  |  |  |  |  |  |  |  |  |  |  |  |  |  |
| weight | G | G | G | G |  | D | D | D | D |  | G2 | G2 | G2 | G2 |
| score | θ_X_ | θ_X_ | θ_X_ | θ_X_ |  | θ_X_ | θ_X_ | θ_X_ | θ_X_ |  | θ_X_ | θ_X_ | θ_X_ | θ_X_ |
| base | Alpha | Theta | Omega | Rho |  | Alpha | Theta | Omega | Rho |  | Alpha | Theta | Omega | Rho |
| sample size | mean | | | |  | mean | | | |  | mean | | |  |
| 25 | 0,803 | 0,817 | 0,879 | 0,899 |  | 0,755 | 0,771 | 0,856 | 0,877 |  | 0,878 | 0,890 | 0,917 | 0,937 |
| 50 | 0,841 | 0,831 | 0,896 | 0,908 |  | 0,800 | 0,806 | 0,879 | 0,888 |  | 0,911 | 0,915 | 0,936 | 0,945 |
| 100 | 0,841 | 0,844 | 0,899 | 0,906 |  | 0,799 | 0,802 | 0,879 | 0,885 |  | 0,915 | 0,918 | 0,939 | 0,944 |
| 200 | 0,841 | 0,843 | 0,899 | 0,903 |  | 0,802 | 0,804 | 0,879 | 0,883 |  | 0,916 | 0,918 | 0,939 | 0,942 |
| Total | 0,831 | 0,834 | 0,893 | 0,904 |  | 0,789 | 0,796 | 0,873 | 0,883 |  | 0,905 | 0,910 | 0,933 | 0,942 |
| N | 1440 | 1440 | 1440 | 1418 |  | 1440 | 1440 | 1440 | 1426 |  | 1440 | 1440 | 1440 | 1418 |
|  |  |  |  |  |  |  |  |  |  |  |  |  |  |  |
|  | Alpha | Theta | Omega | Rho |  | Alpha | Theta | Omega | Rho |  | Alpha | Theta | Omega | Rho |
| sample size | Std. Deviation | | | |  | Std. Deviation | | | |  | Std. Deviation | | |  |
| 25 | 0,106 | 0,107 | 0,049 | 0,051 |  | 0,142 | 0,143 | 0,058 | 0,062 |  | 0,053 | 0,051 | 0,036 | 0,031 |
| 50 | 0,086 | 0,093 | 0,036 | 0,038 |  | 0,118 | 0,120 | 0,045 | 0,048 |  | 0,027 | 0,028 | 0,021 | 0,019 |
| 100 | 0,082 | 0,083 | 0,032 | 0,035 |  | 0,119 | 0,120 | 0,043 | 0,047 |  | 0,020 | 0,021 | 0,017 | 0,016 |
| 200 | 0,078 | 0,079 | 0,030 | 0,032 |  | 0,112 | 0,113 | 0,039 | 0,042 |  | 0,017 | 0,018 | 0,016 | 0,015 |
| Total | 0,090 | 0,092 | 0,038 | 0,040 |  | 0,125 | 0,125 | 0,048 | 0,050 |  | 0,036 | 0,034 | 0,026 | 0,021 |
|  |  |  |  |  |  |  |  |  |  |  |  |  |  |  |

|  |  |  |  |  |  |  | |  |  |  |  | |  | |  |  |  |
| --- | --- | --- | --- | --- | --- | --- | --- | --- | --- | --- | --- | --- | --- | --- | --- | --- | --- |
| weight factor | D2 | D2 | D2 | D2 |  | RAC | | RAC | RAC | RAC |  | | EAC | | EAC | EAC | EAC |
| score | θ_X_ | θ_X_ | θ_X_ | θ_X_ |  | θ_X_ | | θ_X_ | θ_X_ | θ_X_ |  | | θ_X_ | | θ_X_ | θ_X_ | θ_X_ |
| base | Alpha | Theta | Omega | Rho |  | Alpha | | Theta | Omega | Rho |  | | Alpha | | Theta | Omega | Rho |
| sample size | mean | | |  |  | mean | | | | |  | | mean | | | | |
| 25 | 0,853 | 0,866 | 0,902 | 0,923 |  | 0,873 | | 0,885 | 0,914 | 0,934 |  | | 0,897 | | 0,907 | 0,928 | 0,947 |
| 50 | 0,891 | 0,896 | 0,924 | 0,933 |  | 0,899 | | 0,903 | 0,928 | 0,938 |  | | 0,907 | | 0,912 | 0,934 | 0,943 |
| 100 | 0,895 | 0,898 | 0,927 | 0,932 |  | 0,899 | | 0,902 | 0,928 | 0,935 |  | | 0,903 | | 0,906 | 0,931 | 0,937 |
| 200 | 0,897 | 0,899 | 0,927 | 0,931 |  | 0,896 | | 0,897 | 0,926 | 0,930 |  | | 0,898 | | 0,900 | 0,927 | 0,931 |
| Total | 0,884 | 0,890 | 0,920 | 0,930 |  | 0,891 | | 0,897 | 0,924 | 0,934 |  | | 0,901 | | 0,906 | 0,930 | 0,939 |
| N | 1440 | 1440 | 1440 | 1426 |  | 1440 | | 1440 | 1440 | 1418 |  | | 1440 | | 1440 | 1440 | 1418 |
|  |  |  |  |  |  |  | |  |  |  |  | |  | |  |  |  |
|  | Alpha | Theta | Omega | Rho |  | Alpha | | Theta | Omega | Rho |  | | Alpha | | Theta | Omega | Rho |
| sample size | Std. Deviation | | | | | | Std. Deviation | | | | |  | | Std. Deviation | | | | |
| 25 | 0,066 | 0,063 | 0,041 | 0,036 |  | 0,055 | | 0,053 | 0,037 | 0,032 |  | | 0,045 | | 0,042 | 0,034 | 0,027 |
| 50 | 0,035 | 0,036 | 0,023 | 0,022 |  | 0,032 | | 0,033 | 0,022 | 0,020 |  | | 0,028 | | 0,029 | 0,021 | 0,019 |
| 100 | 0,028 | 0,029 | 0,018 | 0,018 |  | 0,025 | | 0,027 | 0,017 | 0,016 |  | | 0,022 | | 0,024 | 0,016 | 0,015 |
| 200 | 0,024 | 0,025 | 0,017 | 0,016 |  | 0,023 | | 0,024 | 0,014 | 0,014 |  | | 0,021 | | 0,022 | 0,014 | 0,013 |
| Total | 0,045 | 0,043 | 0,028 | 0,025 |  | 0,038 | | 0,037 | 0,025 | 0,022 |  | | 0,031 | | 0,031 | 0,023 | 0,020 |

(Figure 4.) Average estimates by different DCERs

(Figure 5.) Deviance between sample and population estimates by selected DCERs

(Figure 7.) Range of the highest and lowest estimates of deviance by DCERs

(Figure 9.) Behavior of DCERs by sample size

(Figure 10a.) Behavior of DCERs by test length (df(X)) with binary items

(Figure 10b.) Behavior of DCERs by test length (df(X)) with polytomous items

(Figure 11a) Behavior of DCERs by the test difficulty; coefficients

(Figure 11b) Behavior of DCERs by the test difficulty; difference between sample and population
